# Bacteriomes, cryptic forms and evolution of a common wireworm pest species, *Hypnoidus bicolor*

**DOI:** 10.1111/imb.70054

**Published:** 2026-06-26

**Authors:** Ivan Drahun, Augustine Chukwunta, Adeniyi Ayodele, Ben G. Pilling, Willem G. van Herk, Bryan J. Cassone

**Affiliations:** ^1^ Department of Biology Brandon University Brandon Manitoba Canada; ^2^ Agassiz Research and Development Centre Agriculture and Agri‐Food Canada Agassiz British Columbia Canada; ^3^ Present address: Department of Biological Sciences Brock University St. Catharines Ontario Canada

**Keywords:** bacteriome, click beetle, metagenomics, parthenogenesis, *Rickettsiella*, *Spiroplasma*

## Abstract

Like other insects, coleopterans harbour dynamic bacteriomes that shape core aspects of their life history. The bacteriomes of several wireworm species (Coleoptera: Elateridae) have been described; however, little research has been undertaken to determine the factors that influence their structure and composition. These soil‐dwelling larvae of click beetles are significant agricultural pests in the Canadian Prairies, with the most ubiquitous species, *Hypnoidus bicolor*, delineated into two genetically distinct clades and both sexual and parthenogenetic populations. In this study, we collected 69 *H. bicolor* adults and larvae from nine populations spanning three Prairie provinces and subjected them to Sanger and 16S rRNA gene sequencing to determine their clade and characterize their bacteriome, respectively. Combined with long‐term surveillance, we provide compelling evidence that the parthenogenetic and sexual populations are associated with different clades. Development, sampling location and host genetics all contributed to the plasticity of *H. bicolor* bacteriomes. These differences are largely attributed to gut bacterial community composition of larvae, whereas, in adults, they appear driven by overall community structure as well as differences in the presence/absence of taxa and within‐clade/population variance. Several notable genera emerged from our study, including Alphaproteobacteria and *Rickettsiella* endosymbionts that predominated in the parthenogenetic clade. Incorporation of this research into integrative pest management and reclassification of *H. bicolor* into a cryptic species complex is also discussed. Overall, this study advances our understanding of Elateridae bacteriomes, including factors that contribute to their richness and community composition.

## Graphical abstract

- Wireworms are important agricultural pests, including *Hypnoidus bicolor* in the Canadian Prairies.
- Parthenogenetic and sexual populations of *H. bicolor* appear associated with different molecular clades. Bacterial community composition and structure were influenced by developmental stage, sampling location and host genetics.
- Taxon differing due to these factors was catalogued, and implications to pest management and bacteriome evolution were discussed.

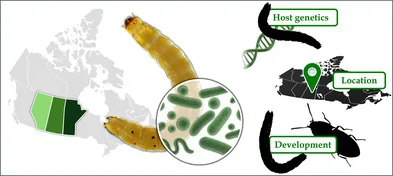

## INTRODUCTION

Coleoptera (i.e., beetles) is the largest order in the animal kingdom, accounting for nearly 40% of described insect species and ~25% of all animals (Stork, 2018). Like other insects, coleopterans harbour gut bacteria symbionts that influence core aspects of their life history (e.g., physiology, ecology and immunity), occurring on a continuum from obligate mutualism to beneficial or pathogenic (Gedling et al., 2018; Paddock et al., 2022; Wielkopolan et al., 2021). Several studies have characterized the bacteriomes of adult beetles (Adams et al., 2013; Ebert et al., 2021; Estes et al., 2013; Magagnoli et al., 2022; Moldovan et al., 2023; Scully et al., 2014) and/or their larval stages (Kucuk et al., 2023; Mohammed et al., 2018; Suárez‐Moo et al., 2020; Wielkopolan et al., 2021), and indicate that the intestinal microbial assemblages are dynamic in nature. A variety of factors contribute to this plasticity, including ontogeny (Suárez‐Moo et al., 2020), pesticide exposure (Giglio et al., 2021), farm management practices (Magagnoli et al., 2022), rearing conditions (Ibarra‐Juarez et al., 2018), host genetics (Paddock et al., 2022), diet and sampling location (Wielkopolan et al., 2021).

Wireworms are the long‐lived larval stages of click beetles (Coleoptera: Elateridae). Many species are globally noted agricultural pests due to the damage (e.g., cosmetic injury, wilting, stunting and death) their feeding activities cause on a variety of economically important crops (Rashed & van Herk, 2024; Traugott et al., 2015). Heavy infestations can result in bare patches in fields and significantly reduced yields, and populations have increased in recent years by the phase‐out of several effective broad‐spectrum insecticides (Barsics et al., 2013; Drahun et al., 2023; Parker & Howard, 2002; Ritter & Richter, 2013; van Herk & Vernon, 2014). Current control typically relies on integrated strategies that indirectly decrease wireworm numbers by targeting the adult stages (e.g., attract‐and‐kill, mass trapping, mating disruption), lessen larval feeding activities for long enough periods to allow for the early season crop to establish, and/or reduce overall larval numbers (e.g., broflanilide, crop rotation, cultivation, entomopathogenic microorganisms) (Brandl et al., 2017; Labrie et al., 2020; Ritter & Richter, 2013; van Herk, Vernon, Goudis, et al., 2021; van Herk et al., 2022, 2023; Vernon et al., 2016; Vernon & van Herk, 2022). Longer term, new avenues for environmentally friendly sustainable wireworm management may include genetic control (e.g., RNAi), formulation of microbial insecticides, and the development of plant‐incorporated protectants and semiochemicals (Qadri et al., 2020).

The first report of bacteria harboured by wireworms was in the 1950s, with the identification of several species of Eubacteriineae from *Melanotus caudex* (Yoshida & Yoshii, 1959). A variety of bacterial flora were subsequently and selectively isolated from *Limonius californicus* and *Agriotes* spp. (Danismazoglu et al., 2012; Kabaluk et al., 2017; Kleespies et al., 2012; Leclerque et al., 2011; Schuster et al., 2013; Zacharuk, 1973a, 1973b, 1974). Lacey et al. (2007) isolated and catalogued various bacterial taxa from the guts of *L*. *canus*, which consisted most notably of *Bacillus*, *Rahnella*, *Paenibacillus* and *Pseudomonas*. More recently, we applied 16S rRNA sequencing to characterize the bacteriomes of five important pests present in the Canadian Prairies: *Hypnoidus bicolor*, *H. abbreviatus*, *L. californicus*, *Aeolus mellillus* and *Dalopius* spp. (Drahun et al., 2025). Over 400 bacterial genera were identified, with each wireworm species harbouring nine or fewer genera that comprised >80% of their gut flora. Further, the plasticity of the wireworm bacteriomes was evidenced by microbial signatures influenced by species and development (early vs. late‐stage larvae). However, this study was unable to provide key insights into the bacteriome dynamics of these coleopterans, as sampling was limited to larvae from a single sampling locality.

Agricultural surveys indicate that *Hypnoidus bicolor* (Eschscholtz 1829) is the primary wireworm species active in early spring in the Canadian Prairie province of Manitoba (>90% of samples), and to a lesser extent in Alberta (47%) and Saskatchewan (44%) (Drahun, Wiebe, Gohl, et al., 2021; van Herk, Vernon, Labun, et al. 2021). Despite its relatively small size, *H. bicolor* is considered an under‐recognized pest species of soybean and potentially other important crops grown in the region (Drahun et al., 2024). Recently, the species was delineated into two distinct clades (A and B), based on sequence divergence of the mitochondrial encoded cytochrome c oxidase subunit I (*COX1*) gene (Drahun, Wiebe, Koloski, et al., 2021). Indeed, the clades show sequence divergence (>4.66%) well above the threshold recommended for species diagnostics using this gene (Hebert et al., 2003). There is some evidence of subtle morphological differences pertaining to setae arrangement within *H. bicolor* (Stibick, 1978, 1979–1980), though its association (if any) with the clades remains unresolved. In addition, putatively sexual and parthenogenetic forms of the species have been identified in Alberta and Saskatchewan based on the presence/absence of males, which appear to overlap the distributions of clades A and B, respectively (Drahun, Wiebe, Koloski, et al., 2021; van Herk & Vernon, 2014; Zacharuk, 1958a, 1958b). It is conceivable that the absence of males in these populations is associated with bacterial flora rather than a form of asexual reproduction, as endosymbiont‐induced parthenogenesis is thought to occur in ~40% of arthropod species having sex ratio distortion (van der Kooi et al., 2017). In addition, bacterial‐mediated cytoplasmic incompatibility (Nor et al., 2013), male‐killing (Hurst & Jiggins, 2000) and feminization (Kageyama et al., 2002) can result in exclusively or predominantly female individuals in a given insect population/species.

The *H. bicolor* bacteriome has been characterized for mtDNA clade A, from a sampling location in Manitoba, Canada (Drahun et al., 2025). The researchers identified 105 genera harboured by the wireworm. However, nothing is currently known regarding the influence of ontogeny (larvae vs. beetle), host genetics (clade A vs. B) and sampling location on the bacterial richness/evenness, community composition and structure of *H. bicolor* or any other wireworm/click beetle species. With this in mind, the objective of our study was to perform 16S rRNA gene amplicon sequencing and partial mitochondrial COX1 gene sequencing to characterize the bacteriomes of *H. bicolor* adults and larvae clade A and B collected from multiple agricultural fields across the Canadian Prairies.

## MATERIALS AND METHODS

### Wireworm and beetle sampling

Wireworms were collected in 2020 from four agricultural fields in Manitoba, with the following geographical coordinates and the closest location (approx.) and field ID in parentheses: 50.2532, −100.8972 (Hamiota; HS); 50.041246, −100.055400 (Rivers; ES); 49.166178, −99.313315 (Neelin; MY); and 49.6377, −99.5654 (Treesbank; MS). We sampled in April and May using a standardized bait trapping procedure, as previously described (Drahun, Wiebe, Gohl, et al., 2021; Drahun et al., 2022). Briefly, 10.2 cm plant pots served as bait traps and consisted of 100 mL of untreated hard red spring wheat seed in between two layers of coarse vermiculite. Prior to placement in a field, germination was initiated by soaking each trap twice. In the field, we dug a 15 cm circular hole, removed adjacent vegetation, broke up the underlying soil and placed the trap snugly within. The trap was then covered with 2.5 cm of soil, a 20 cm diameter inverted plastic tray was placed on top, and a stake flag was used to mark the location. Trapping was done on the field peripheries, with a total of 16 traps per field positioned ~5 m apart in linear transects of four traps per transect. After ~10 days in the soil, we collected the traps and extracted the wireworms using Tullgren funnels.

We sampled adult female beetles in May of 2019 from five fields encompassing Manitoba, Alberta and Saskatchewan: 49.8407, −110.7215 (Medicine Hat, AB; PZ); 49.1047, −97.5596 (Altona, MB; PY); 50.4452, −104.6188 (Regina, SK; X); 50.0333, −113.5852 (Claresholm AB; V); and 53.0216, −108.2736 (Meota, SK; U). Beetles were collected using Vernon Pitfall Traps™ (Intko Supply Ltd), as described in Vernon and van Herk (2017). *Hypnoidus bicolor* individuals were identified using morphology‐based reference keys (Brooks, 1961; Etzler, 2013; Glen et al., 1943). All samples (beetle and larvae) were surface sterilized using Tween® 80 per 10 mL of 0.5% bleach, 0.5% benzalkonium chloride and 75% ethanol. We then stored the samples in 75% ethanol at −20°C.

### DNA isolation and 16S rRNA gene sequencing

Each specimen was placed in a sterilized Petri dish, and the digestive tract was excised from the surrounding tissues via a lengthwise ventral incision using a new set of sterilized scalpels (size 10 blades) and forceps per individual. We used the One‐4‐All Genomic DNA Miniprep Kit (Bio Basic, Markham, ON) to extract gDNA from each sample (i.e., an individual wireworm or beetle) as well as five negative controls that were processed identically but contained lysis buffer without animal tissues. The DNA was quantified and assessed for quality using the Nanophotometer NP80 (Implen Inc., Westlake Village, CA). We amplified the 16S rRNA gene (V4 region) in 40 μL reactions using Phusion High‐Fidelity PCR Master Mix with HF Buffer (New England Biolabs, Ipswich, MA) and the primer set: 515F: GTGCCAGCMGCCGCGGTAA and 806R: GGACTACHVGGGTWTCTAAT. For downstream sample identification and multiplexing, the primer sets were adapter‐tagged (FwR1: ACACTCTTTCCCTACACGACGCTCTTCCGATCT; RvR2: GTGACTGGAGTTCAGACGTGTGCTCTTCCGATCT). We performed the PCRs on the samples (product size ~300 bp) and negative controls using a Biometra TOne thermal cycler (Analytik Jena AG) and the thermal parameters detailed in Boelsen et al. (2019): 98°C for 30 s, then 35 cycles of 98°C for 5 s (denaturation), 65°C for 20 s (annealing) and 72°C for 15 s (elongation). A final extension was done at 72°C for 5 min followed by 4°C until removal. Library barcoding, quality assessment and paired‐end sequencing (250 bp) of the adapter‐tagged amplicons was done at Génome Québec Innovation Centre (McGill University, Montreal, QC, Canada) using the BioAnalyzer 2100 (Agilent Technologies, Santa Clara, CA) and Illumina MiSeq platform. The raw sequence reads are available from the NCBI short sequence read archive (SRA) under the accession number PRJNA1100600 (SUB14380285) (Cassone, 2026). Available from: https://www.ncbi.nlm.nih.gov/sra.

### Clade diagnostics

For each sample subjected to 16S rRNA gene sequencing, we also performed mtDNA clade (A or B) diagnostics, as described in Drahun, Wiebe, Koloski, et al. (2021). In brief, a 710‐bp fragment of the mitochondrial cytochrome c oxidase subunit I (*COX1*) gene was amplified for the beetle samples using the universal primer set described in (Folmer et al., 1994): LCO1490: 5′‐GGTCAACAAATCATAAAGATATTG‐3′ and HC02198: 5′‐TAAACTTCAGGGTGACCAAAAAATCA‐3. The PCRs were done in 20‐μL reactions with Platinum SuperFi PCR Master Mix (Invitrogen, Waltham, MA): 95°C for 3 min followed by 35 cycles of 95°C for 20 s, 50°C for 20 s, 72°C for 30 s, and a final elongation of 72°C for 5 min. PCR products were run for 45 min at 100 V on 1.5% agarose gels stained with ethidium bromide and visualized via a ChemiDoc Imaging System (Bio‐Rad Laboratories, Hercules, CA). Amplified PCR products were sent to Génome Québec for purification using a Biomek NX robot with a bead solution and Sanger sequencing of the forward strand with the 3730xl DNA Analyzer (Applied Biosystems, Westminster, CO).

Each resultant *H. bicolor* sequence was assigned to a given clade, and specifically a haplotype within the clade, using the nucleotide BLAST (BLASTn) function on NCBI (https://blast.ncbi.nlm.nih.gov/), with all 39 *COX1* mtDNA haplotype sequences identified in Drahun, Wiebe, Koloski, et al. (2021) inputted as the subject sequences.

### Data analysis

Raw paired‐end reads were imported into QIIME2 v2026.4 (Bolyen et al., 2019) using the input format “PairedEndFastqManifestPhred33V2” through the manifest file. Adapter sequences, including 16S rRNA gene primers (515F/806R), were removed using Cutadapt v5.3 (Martin, 2011), and the following parameters: minimum length = 50 bp and error rate = 0.1. Sequence quality was assessed using demux summary and visualized via Qiime2view (https://view.qiime2.org/). Based on the quality profile, where scores declined below 25, forward and reverse reads were each truncated at a length of 220 bp. The sequence reads were then subjected to denoising via DADA2 v1.36.0 (Callahan et al., 2016) to generate amplicon sequence variants (ASVs). The resultant ASVs were screened for contaminants using decontam v3.12 (Davis et al., 2018). By assigning a probability threshold of 0.1, potential contaminants were identified based on ASV prevalence in the negative controls versus true samples. The contaminants and negative controls were simultaneously filtered out from the ASV table and representative sequences. For taxonomic classification, we used a pre‐trained Naive Bayes classifier database (SILVA 138, 99%; Quast et al., 2013). The ASV table was then filtered to exclude chloroplast, mitochondria, and those that remained unassigned at the phylum level. Similarly, the representative sequences were filtered to match the ASV table.

Next, we imported the ASV tables and associated metadata into the Marker Data Profiling tool in MicrobiomeAnalyst 2.0 (Chong et al., 2020; Dhariwal et al., 2017) to evaluate the diversity indices. Data with one sample occurrence was omitted, and variance and low count filters were set at 10% inter‐quantile range and 20% prevalence, respectively. Rarefication was based on the minimum library size after filtration: 1498 (all data), 3342 (beetle) and 4279 (wireworm). We implemented total sum scaling (TSS) to bring the sample data to the same scale. Alpha diversity indices are a good indicator of bacterial richness and evenness and were calculated as Shannon Entropy (Hughes et al., 2001; Lozupone & Knight, 2008). Alpha diversity was visualized using bar plots and significance was assessed through post hoc Mann–Whitney/Kruskal–Wallis pairwise tests with multi‐testing (FDR) adjustment based on Benjamini–Hochberg (*p* < 0.05). Beta diversity provides insights into the composition and structure of bacterial communities, and was measured using Bray–Curtis (Bray & Curtis, 1957) and, where applicable, Jaccard similarity index (Baselga & Orme, 2012). We constructed two‐dimensional Principal Coordinates Analysis (PCoA) plots to display the computed beta diversity distance matrices. Significance was determined via permutational multivariate analysis of variance (PERMANOVA) and permutational multivariate analysis of dispersion (PERMDISP) at FDR adjusted significance thresholds of *p* < 0.05. Network correlation analyses were also done for bacteria at the genus level for beetles and wireworms using sparse correlations for compositional data (SparCC; Friedman & Alm, 2012) with the following parameters: 200 permutations, correlation threshold ≥0.5, and *p* < 0.05 (default setting herein).

To explore differences in the beetle and wireworm bacteriomes, we implemented analysis of compositions of microbiomes with bias correction 2 (ANCOM‐BC2) using the ANCOMBC v2.14.0 (Lin & Peddada, 2020) and tidyverse v2.0.0 (Wickham et al., 2019) libraries in R (R Core Team, 2025). The ASV tables were filtered to the genus level for these analyses, or the next hierarchical classification in cases where genus could not be resolved. Moreover, we performed ANCOM‐BC2 globally for development (beetle vs. wireworm) and clades (A vs. B), and both globally and pairwise for population. Default parameters were implemented for multiple testing correction and feature filtering. In accordance, taxa with a prevalence less than 10% (prv_cut = 0.10) were omitted from the analyses. Resultant p‐values were adjusted for multiple comparisons using the Benjamini–Hochberg method (p_adj_method = “BH”) with a false discovery rate (FDR) threshold of *q* < 0.05. Variance regularization was applied using a small positive constant (s0_perc = 0.05) and structural zero detection was disabled (struc_zero = FALSE). Output files were imported/exported as .xls files using the libraries readxl and writexl (Wickham & Bryan, 2026). To visualize the effect sizes and significance of the ANCOM‐BC2 statistical models, log‐fold changes and their corresponding adjusted q‐values were plotted using the ggplot2 v4.0.3 package (Wickham, 2016) in R. Finally, Pearson correlation coefficient was calculated to determine whether the number of significant genera was associated with geographical distance between localities for both beetles and wireworms (*p* < 0.05).

## RESULTS

### Clade diagnostics

Figure 1 displays the sampling localities for *H. bicolor* in the Canadian Prairies, which includes the relative proportions of clade A and B, and number of individuals collected and subjected to clade diagnostics at each site. The five beetle populations were designated as sexual or parthenogenetic based on the presence/absence of males via long‐term surveillance efforts, as previously described (Drahun, Wiebe, Gohl, et al., 2021). Moreover, the genetic relatedness of the clades is visualized in a previously published median‐joining network (Drahun, Wiebe, Koloski, et al., 2021). All individuals from the two parthenogenetic populations (U and V) were clade B and corresponded to haplotype 8 (58%) or 12 (42%). For the sexual populations, all beetles in PY were clade A, whereas both clades were present in X (87% clade A) and PZ (78% clade A). The haplotypes identified from clade A beetles were 1 (29.2%), 4 (58.3%), 9 (8.3%) and 23 (4.2%). All four wireworm populations were sampled from the same geographical region (West Manitoba), and both clades were present in each at roughly equal ratios. Haplotypes 1, 4 and 9 predominated for clade A, whereas 8 and 12 were the most represented for clade B.

**FIGURE 1:**
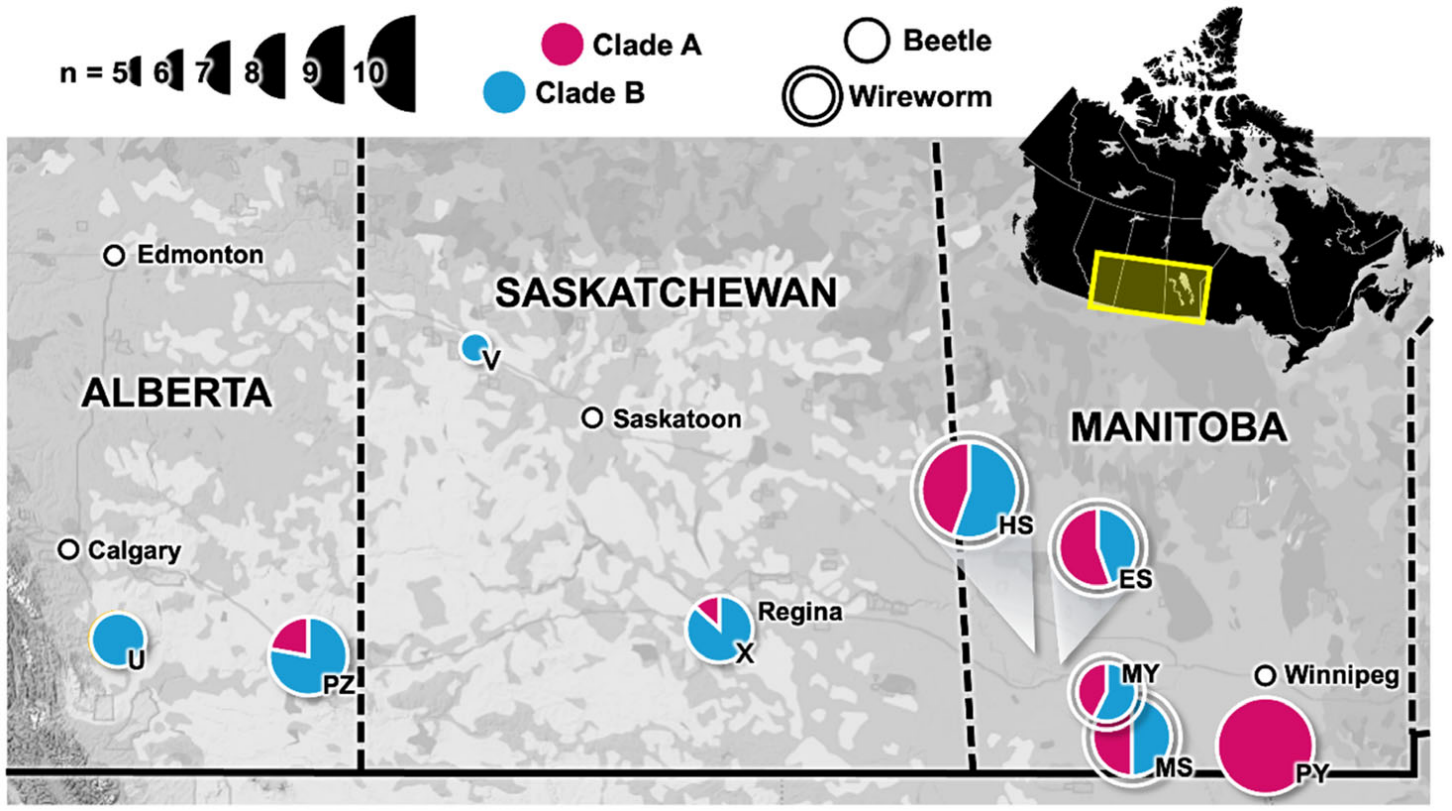
The nine sampling localities for *Hypnoidus bicolor* adults and larvae in the Canadian Prairies. Relative abundances of the mtDNA clades (a and b) in each locality are shown in the pie charts.

### Sequencing and taxonomic breakdown

We sequenced a subset of 69 *H. bicolor* samples from four wireworm and five beetle populations (Table S1). Each sequenced wireworm population consisted of individuals from both clades A and B, whereas we sequenced exclusively A or B individuals from each beetle population. This is due to the absence of one of the clades in a given population or sample sizes too small for replication. All derived sequences were from individual specimens (i.e., not pooled samples), and the larvae (9–9.3 mm) and beetles (~5.6 mm) selected were of similar sizes. Further, only female beetles were included in the study (wireworms could not be resolved to sex). We generated a total of 3,959,408 paired‐end reads, of which ~65% were from the beetle samples. After filtering by quality, between 74% and 92% of reads remained per sample. Subsequent denoising, read merging, and removal of chimeric reads yielded 55% to 88% of input reads remaining for a given sample. After filtering for contamination and unassigned taxa, a combined total of 1440 ASVs were identified across the wireworm and beetle datasets, including 1117 and 402 for the wireworm and beetle subsets, respectively. These ASVs were further delineated into 198 beetle and 348 wireworm genera, or next hierarchical group if genus could not be revolved.

Taxonomic classification identified 11 phyla, which were represented in both beetles and wireworms. Proteobacteria was the most populous phylum for adults (84.7% of reads) and larvae (69.6%). Actinobacteriota (20.7%) and Bacteroidota (6.8%) were found in considerably higher proportions for wireworms than for beetles (<0.04% for each). In turn, Firmicutes was the second most populous phylum harboured by beetles (14.7%) and comparatively devoid in wireworms (0.7%). All other phyla were represented in low proportions in both developmental stages (<0.8%).

### Factors influencing the *Hypnoidus bicolor* bacteriome

The amplicon sequencing data was analyzed via three approaches to provide insights into the influences of development, sampling location and clade on the bacteriomes, as detailed below.

#### Development

As a first step, we assessed differences in the bacteriomes between the adult and larval stages. Alpha diversity was significantly higher for wireworms than beetles (*p* < 0.001; Figure 2a). Beta diversity differed between developmental stages for PERMANOVA (*F*‐value = 10.74; *p* = 0.001; Figure 2b), but not for PERMDISP (*F*‐value = 0.84; *p* = 0.362). This indicates that adults and larvae have distinct microbial communities, and this difference is not confounded by how variable the microbiomes are within each life stage (i.e., equal dispersion). Figure 2c displays the most represented genera for beetles and wireworms. In total, 92 genera (or the next highest classification in cases where genus could not be determined) significantly differed (*q* < 0.05) between developmental stages (Figure 2d).

**FIGURE 2:**
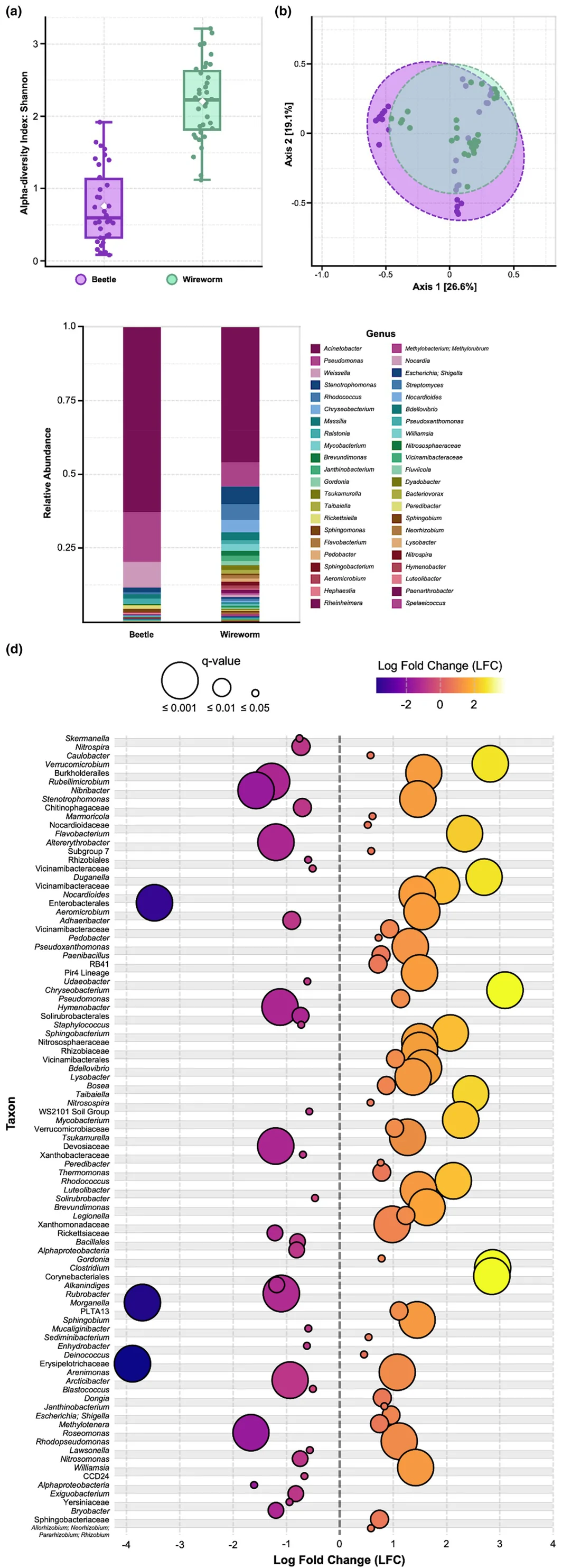
The influence of development (adults and larvae) on the bacteriomes of *Hypnoidus bicolor*. (a) Boxplots displaying Shannon Entropy alpha diversity for the bacterial amplicon sequence variants (ASVs); (b) principal coordinate analysis (PCoA) of bacterial ASVs constructed using Bray–Curtis distance matrices; (c) relative abundances of the most common bacterial genera harboured at each developmental stage; and (d) dot plot of significant taxon identified by ANCOM‐BC2. The x‐axis represents log fold change, showing the magnitude and direction of difference for each taxon (visualized on a colour scale). The size of the circle corresponds to the statistical significance (q‐value).

#### Sampling location

Given that the adult and larval bacteriomes differed considerably from one another, we carried out the subsequent analyses on the developmental stage datasets independently. Figure 3 shows location‐based box and PCoA plots of alpha and beta diversity, respectively. There were no differences in alpha diversity between sampling sites for both beetles and wireworms. Beta diversity indices for the beetle ASVs indicated both PERMANOVA (*F*‐value = 5.29; *p* = 0.001) and PERMDISP (*F*‐value = 3.93; *p* = 0.011) were significant across sampling localities. Both metrics being significant indicates that we cannot confidently conclude that geographic differences are solely driving bacterial community composition, as the results are fundamentally confounded by dispersion effects. To this end, we implemented Jaccard index (presence‐absence metric), which was also significant for PERMANOVA (*F*‐value = 4.34; *p* = 0.001). This means that the localities differ by which bacterial assemblages are present or absent in a given locality, rather than just shifts in their relative abundances. All post hoc pairwise comparisons were significant except for V vs. PZ (*p* = 0.159) and V vs. U (*p* = 0.128). A total of 74 genera significantly differed among localities (Table S2). Moreover, between 2 and 30 genera differed between pairwise locations, with results of the Pearson correlation indicating a significant positive relationship between the number of significant genera identified and geographic distance between localities (*R* ^2^ = 0.698; *p* = 0.025) (Figure S1).

**FIGURE 3:**
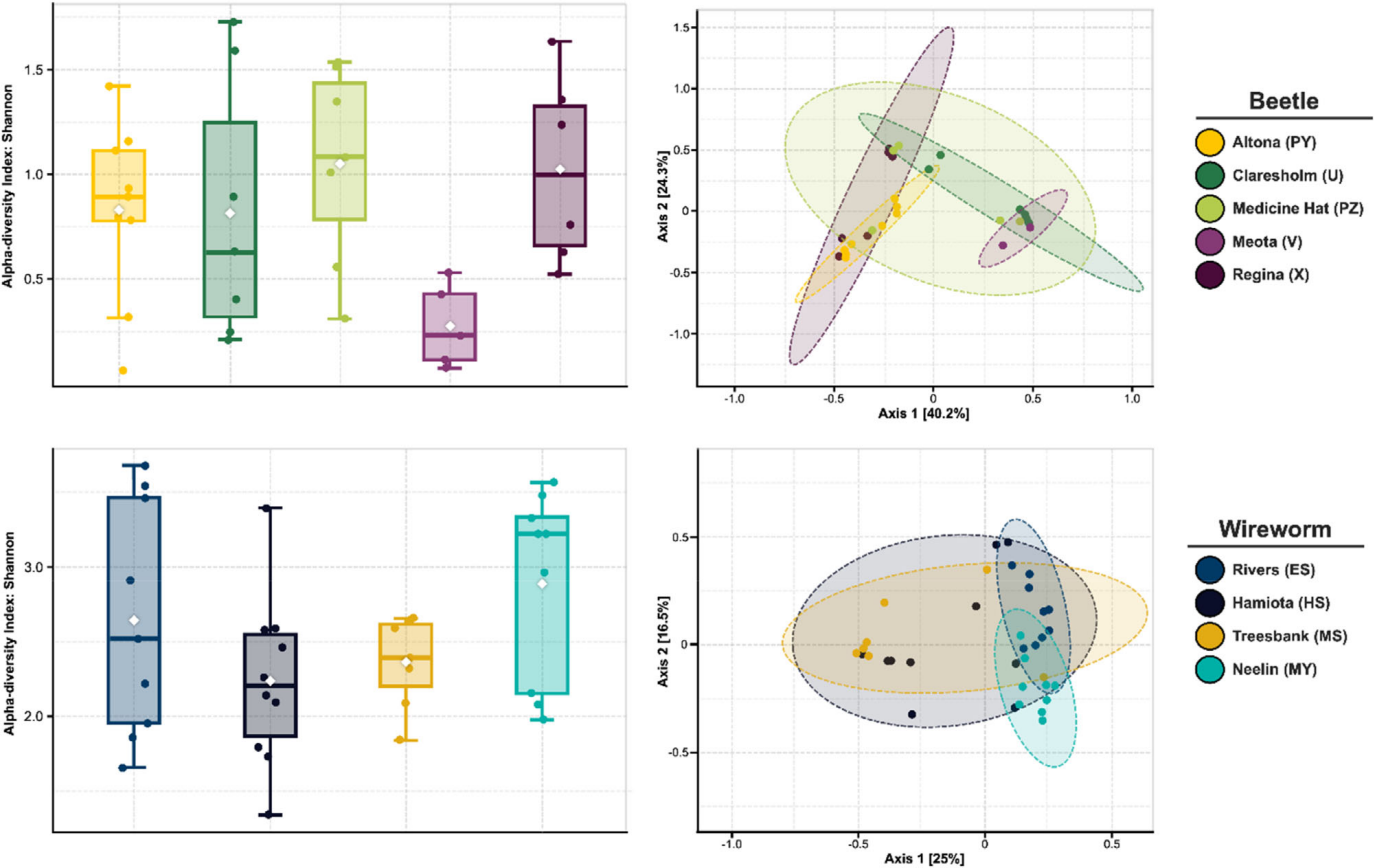
Shannon entropy alpha diversity and Bray–Curtis distance matrices indices for the bacterial amplicon sequence variants (ASVs) across sampling localities of adult and larval *Hypnoidus bicolor*. Alpha diversity and beta diversity are visualized as box plots and PCoA plots, respectively.

For wireworms, beta diversity differed between populations for PERMANOVA (*F*‐value = 3.51; p = 0.001), but not for PERMDISP (*F*‐value = 0.73; *p* = 0.544). Beta diversity was significantly different across all post hoc pairwise locations for the larvae (*p* < 0.01, all contrasts), with one exception: HS and MS (*p* = 0.292). A total of 111 genera significantly differed among localities (Table S2), including between 4 and 19 pairwise locations. Results of the Pearson correlation indicated that there is a non‐significant negative relationship between the number of significant genera identified and geographic distance between localities (*R* ^2^ = 0.576, *p* = 0.232) (Figure S1).

Figure 4a shows the relative abundances of bacterial genera harboured by adults and larvae across the sampling localities. Network analysis revealed considerable associations among the bacteria, which may be indicative of functional synergies. Specifically, the genera represented in each network exhibited similarities in patterns of relative abundance across multiple localities. The primary network consisted of >40 genera (Figure 4b), with five smaller networks comprised of 2–3 genera each. Only two small networks were identified for the beetles: one comprising *Acinetobacter*, *Weissella*, *Ralstonia* and *Stenotrophmonas*, and another with *Pseudomonas* and *Methylobacterium/Methylorubrum*.

**FIGURE 4:**
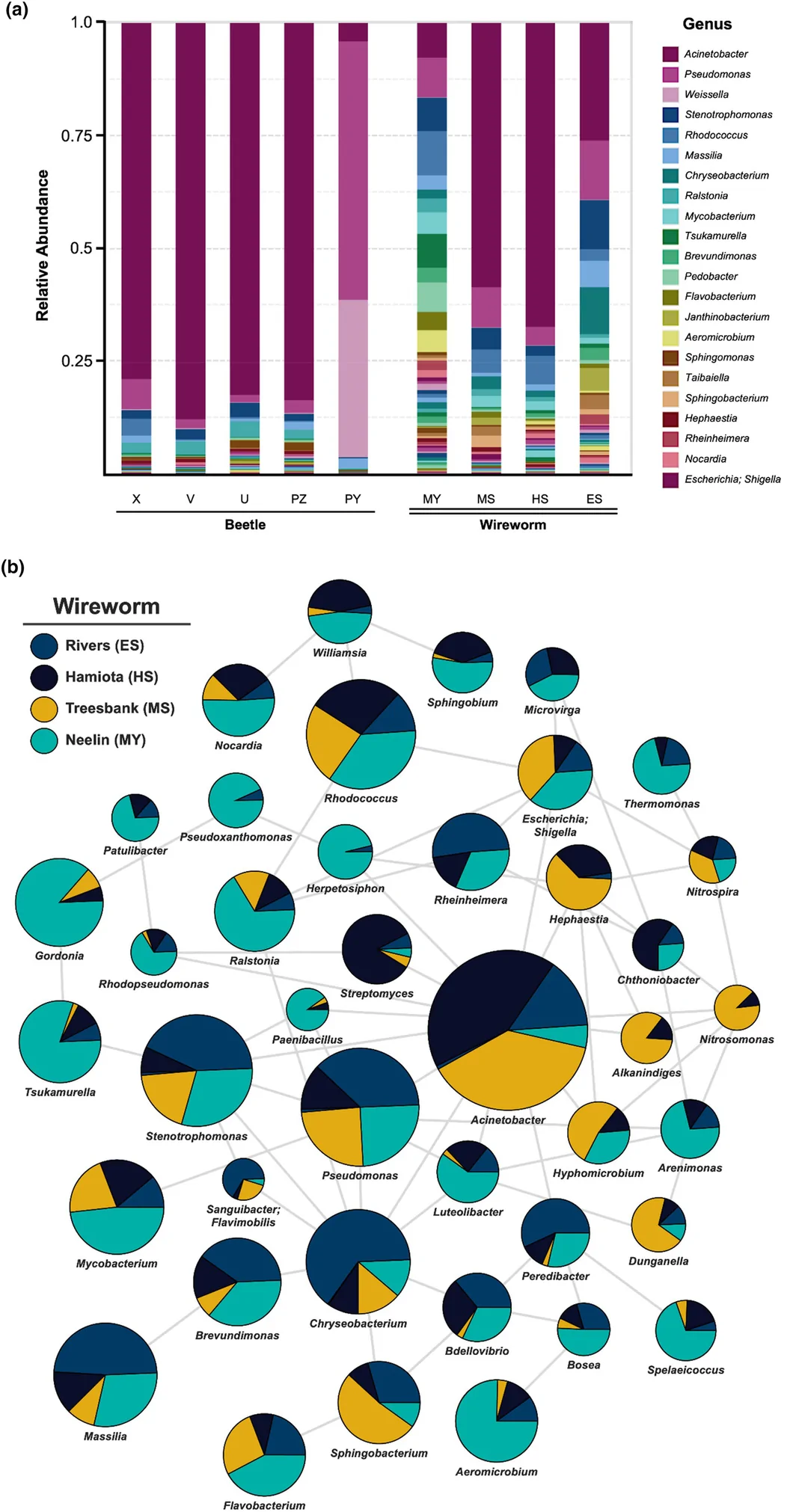
The impact of sampling locality on the bacteriomes of *Hypnoidus bicolor*. (a) Abundances of the most common bacterial genera across localities; (b) genus‐level network analyses of the bacteriomes. Associations among wireworm genera were derived using SparCC and a threshold for significance of *r* ≥ 0.5 and *p* < 0.05.

#### Clade

As *H. bicolor* is delineated into two genetically distinct mtDNA clades (A and B), we next endeavoured to determine if non‐random associations exist between bacteria and clade across our sampling range. Alpha diversity was significantly higher in clade A than B for beetles (*p* = 0.034; Figure 5a). There were no differences in alpha diversity between clades for the wireworms (*p* = 0.525; Figure 5b). For the adults, beta diversity differed between clades for PERMANOVA (*F*‐value = 10.12; *p* = 0.001; Figure 5c) and PERMDISP (*F*‐value = 36.21; *p* < 0.001). Jaccard Index of PERMANOVA was also significant (*F*‐value = 8.3; *p* = 0.001). Beta diversity of the larvae differed between clades for PERMANOVA (*F*‐value = 3.41; *p* = 0.004; Figure 5d), but not for PERMDISP (*F*‐value = 0.8; *p* = 0.378).

**FIGURE 5:**
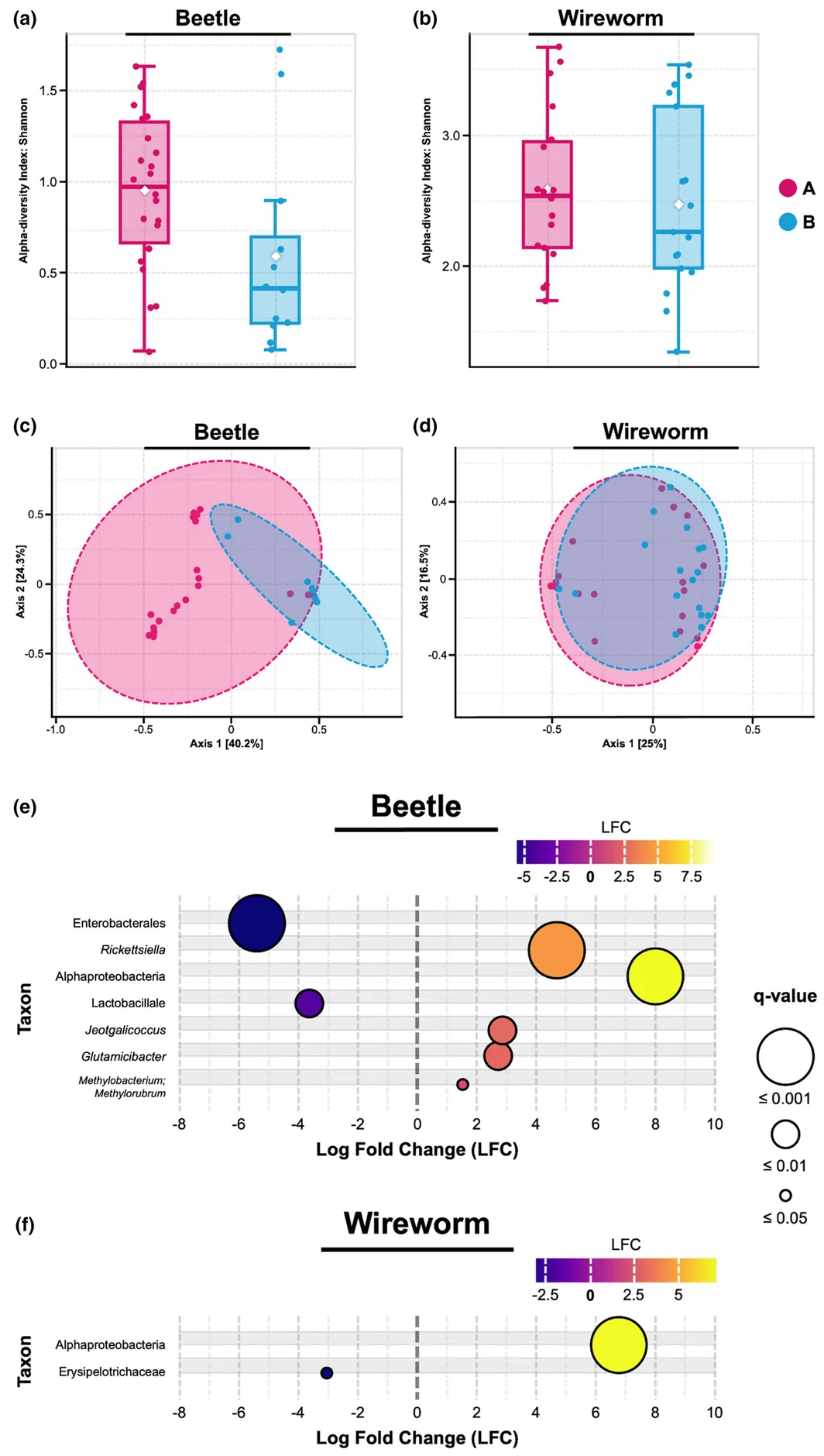
The influence of host genetics (mtDNA clade a and b) on the bacteriomes of *Hypnoidus bicolor* beetles and larvae. (a, b) Boxplots displaying Shannon Entropy alpha diversity for the bacterial amplicon sequence variants (ASVs); (c, d) Principal coordinate analyses (PCoA) of bacterial ASVs constructed using Bray–Curtis distance matrices; (e, f) Dot plots of significant taxa identified by ANCOM‐BC2. The x‐axis represents log fold change, showing the magnitude and direction of difference for each taxon (visualized on a colour scale). The size of the circle corresponds to the statistical significance (*q*‐value).

Figure 5e,f displays the genera found in significantly higher relative abundance in clade A or B for beetles and wireworms, respectively. For the adults, seven genera significantly differed between clades (*q* < 0.05). Notable were *Rickettsiella* and *Glutamicibacter*, as these were present in all clade B samples and largely devoid from clade A. Also enriched in clade B were *Jeotgalicoccus*, *Methylobacterium‐Methylorubrum* and Alphaproteobacteria (unknown genus). On the other hand, unknown genera belonging to Lactobacillales and Enterobacterales were enriched in clade A. Only two unknown genera significantly differed for wireworms: Alphaproteobacteria (clade B) and Erysipelotrichaceae (clade A).

### Resident microbiome

Although the structure and composition of the insect microbiome is influenced by a variety of factors, we next explored whether *H. bicolor* harboured genera that were consistently present during development and across different geographical locations. Only *Acinetobacter* was identified in all 69 samples, suggesting it represents a more permanent fixture of the microbiome. In addition, *Pseudomonas*, *Ralstonia*, *Stenotrophomonas* and Burkholderiales (unknown genus) were present in >90% of samples, including both beetles and wireworms and all sampling localities.

## DISCUSSION

Host‐microbe associations undoubtedly impact core aspects of insect life history; however, little research has been undertaken to advance our understanding for wireworms or, in general, coleopterans. Therefore, the aim of our study was to assess the impact development, sampling location and host genetics have on the bacteriomes harboured by a notable pest species in the Canadian Prairies, *H. bicolor*. Development (adults vs. larvae) was a major factor contributing to differences in the bacteriomes of *H. bicolor*, with greater species richness and evenness in wireworms and extensive differences in structure and composition between them. Sampling location and host genetics (mtDNA clade A or B) also had a pronounced impact on the bacterial assemblages of both life stages. For wireworms, these differences are largely attributed to microbial community structure; whereas, in beetles, they appear driven by overall community composition in addition to differences in presence/absence of taxa and within‐clade/population variance. It must be emphasized that wireworm/beetle sampling is time‐sensitive, labour‐intensive, largely unpredictable and influenced by a myriad of agro‐environmental factors that are not within the control of the collection strategy. Therefore, limitations in our ability to collect both clades in each beetle locality or both developmental stages from the same localities/year presumably impacted the findings of our study to some extent. Nonetheless, our study indicates development, host genetics and location significantly influence the bacteriomes of *H. bicolor*.

Parthenogenetic and sexual populations of *H. bicolor* have been putatively identified, with geographical distributions that suggest reproductive strategy is associated with mtDNA clade (Drahun, Wiebe, Koloski, et al., 2021; van Herk & Vernon, 2014; Zacharuk, 1958a, 1958b). Our results provide convincing evidence that clade B is parthenogenetic, whereas clade A is sexual. Although both clades overlap in some parts of their range, our diagnostic testing indicated that populations historically believed to be parthenogenetic due to the presence of only female beetles, were all clade B. Most click beetles found in Canada are sexual; however, at least one species, *Aeolus mellillus*, is parthenogenetic (Glen et al., 1943). The reduced alpha diversity of clade B beetles relative to clade A is consistent with that found for *A. mellillus* in comparison to sexual wireworm species (Drahun et al., 2025). Although little has been reported, sex‐biased bacteriomes may constrain species richness for parthenogenetic species (Muratore et al., 2020; Wan et al., 2020). We could not identify any consistent morphological differences between the clades (e.g., setae arrangement); however, this possibility cannot be dismissed as ultrastructural differences can be challenging to resolve. On a broader level, we propose that the discrete genetic, and now bacteriome, differences between clades are sufficient for reclassification as cryptic species within a newly formed *H. bicolor* complex. Cryptic species are common in insects, including agriculture pests (Hendrichs et al., 2015; Li et al., 2023; Walter, 2003) and other coleopterans (Duran et al., 2019). Moreover, wireworm genetic analyses indicated *Limonius californicus* and *L. infuscatus* populations across the northwest United States and southwest Canada are comprised of multiple genetically distinct, putatively cryptic species (Andrews et al., 2020).

Analogous to true parthenogenesis as a form of asexual reproduction (Aguín‐Pombo & Kuznetsova, 2023), endosymbiotic bacteria can induce a preponderance of females in insects via male‐killing (Hurst & Jiggins, 2000), feminization (Kageyama et al., 2002) and cytoplasmic incompatibility (Nor et al., 2013). It is therefore conceivable that some of the variability in bacteriomes between *H. bicolor* clades contribute to their divergent reproductive strategy, particularly if they are maternally inherited, predominate in clade B, and known to cause sex‐ratio distortion. Alphaproteobacteria was among the most significant bacteria identified for both developmental stages, and enriched in clade B. Although we could not resolve the significant bacteria within this class to the genus level, many members are intracellular endosymbionts, and include the notorious male‐killing *Wolbachia* and *Spiroplasma* (Hurst et al., 1999; Hurst & Jiggins, 2000; Jiggins et al., 2000; Arai et al., 2024). Also notable for beetles was *Rickettsiella*, which was identified nearly exclusively in clade B. In aphids, *Rickettsiella* are facultative endosymbionts that affect ecological traits (Tsuchida et al., 2002) and play protective roles against fungal pathogens (Łukasik et al., 2013). Intriguingly, *Rickettsiella* induce strong cytoplasmic incompatibility in a spider host (Rosenwald et al., 2020). While Rosenwald and coauthors showed that infection with these vertically inherited bacteria impacted hatching rates, it is possible that *Rickettsiella* skews sex ratios in some arthropod species via a similar mechanism. Such endosymbiont‐induced cytoplasmic incompatibility has been documented in several coleopteran species (Aikawa et al., 2022; Kiefer et al., 2022; Takano et al., 2017; Wade & Stevens, 1985). It must be emphasized that our findings are correlative and generate hypotheses for future functional validation (e.g., via metagenomics to identify potential virulence genes, or experimental inoculation).

The only other significant clade differences in wireworms was the enrichment of Erysipelotrichaceae in clade A. This family naturally inhabits the intestines of insects, serving as key symbiotic fermenters that may aid in digestion, lipid metabolism and host development (Tegtmeier et al., 2016). More divergence between clades was identified for the beetles, including a preponderance of *Glutamicibacter, Jeotgalicoccus* and *Methylobacterium‐Methylorubrum* in clade B. Members of *Glutamicibacter* can potentially impact host fitness by influencing the gut microbiota (Li et al., 2024). Neither *Jeotgalicoccus* nor *Methylobacterium‐Methylorubrum* are considered primary or specialized insect gut symbiont, with its presence likely transient and diet‐driven (Crippen et al., 2022; Mayoral‐Peña et al., 2022). For clade A, Lactobacillales are noted core members of the bacteriomes of agricultural pests, aiding in host adaptation and survival during dietary shifts (Palacio et al., 2021; Yuan et al., 2021). Enterobacterales are ubiquitous inhabitants of the insect gut microbiome, actively involved in host nutrition, food digestion, detoxification of plant compounds and immune regulation (Jane and Kikuchi, 2020; Viswan & Augustine, 2026). The extent to which any of these taxa impact life history within clades will require future, more targeted research.

Development also had a pronounced influence on the *H. bicolor* bacterial assemblages. Since the life cycle of *H. bicolor* includes a two‐ to three‐year larval period (Catton et al., 2021), we collected wireworms (and beetles) at the same time of year and of similar sizes to minimize temporal variability within a given life stage (Drahun et al., 2025). Nevertheless, the adult and larval bacteriomes have fundamentally different bacterial community compositions. This may be at least partially attributed to transiently present microbes acquired from their diet. Although little research has been undertaken for beetles, lepidopteran (i.e., butterflies and moths) bacteriomes appear largely temporary, where microbes are acquired and maintained through the ingestion of food (Gohl et al., 2022; Hammer & Bowers, 2015; Shao et al., 2024). Wireworms are subterranean feeding on the roots, cotyledons and other accessible plant parts; whereas, click beetle feeding ecology is less understood with some species feeding on nectar, pollen and flowers (Drahun, Wiebe, Gohl, et al., 2021; Serrano et al., 2023; van Herk & Vernon, 2014). Dietary‐driven anatomical changes to the beetle digestive tract can occur during metamorphosis (Chiang & Shelomi, 2023), with the altered gut physiology presumably impacting the subset of microbes that can establish and proliferate. This selectivity may account for the lower alpha diversity observed in beetles, though the oral filter can influence the larval microbiota flora by preventing the ingestion of larger bacteria (Zacharuk, 1973b). Non‐specific feeding behaviours (e.g., ingestion of soil) and core bacteria that are important for a given life stage also likely contribute to ontogeny‐based bacteriome differences. Some of the variability may be due to adults being collected a year prior to the larvae, which likely influenced plasticity to some extent. Indeed, inter‐annual environmental variation (e.g., weather, crop rotation, soil management and pesticide exposure) are likely to fundamentally alter the bacteriomes.

Bacterial communities among wireworm sampling locations were also distinctly structured, likely due to diet (i.e., accessible plants) and the soil microbial profiles. Over 100 genera differed across localities, with each pairwise population comparison showing structural differences. The vast majority of genera were identified at relative abundances <2%, owing to the considerable richness and evenness within. On the other hand, beetle bacteriomes were less taxon‐rich, and differed primarily in taxa present (composition/presence‐absence), rather than relative abundances. For instance, *Weissella* was the second most abundant genus harboured by Altona beetles and largely devoid from the other populations. This genus naturally inhabits the guts of many beetle species as core symbionts, aiding in digestion, nitrogen cycling and fatty acid biosynthesis (Braglia et al., 2025; Valdivia et al., 2023). Other genera were absent from specific locations, including *Cutibacterium*, *Adh*aeribacter and *Mycobacterium*. Moreover, geographical distance appears to be a key factor shaping their bacteriomes, as there was a positive correlation in the number of significant genera between populations and kilometres between them. This process of “distance‐decay” can cause divergent microbial profiles due to diet and resource availability, environmental heterogeneity and dispersal barriers (Finkel et al., 2012; Paddock et al., 2022).

The mechanisms by which coleopterans acquire and maintain their bacteriome remain largely unknown (Drahun et al., 2025). Their bacterial assemblages are undoubtedly influenced by diet (Wielkopolan et al., 2021), but they also appear to host a stable community (Bozorov et al., 2021; Lu et al., 2024). *Acinetobacter* was present in all adult and larval samples, suggesting this genus a member of the resident microbiota. Members of this genus are found in a wide variety of insects and can form a beneficial symbiosis with their host (Li et al., 2022; Tang et al., 2024). Several other genera identified at each developmental stage and in the vast majority of samples (e.g., *Pseudomonas*, *Ralstonia*, *Stenotrophomonas*) are potentially resident microbiota. *Pseudomonas* predominates in several wireworm species (Drahun et al., 2025), and have been proven advantageous to their coleopteran host by detoxifying their microenvironment, providing nutrients and protecting against pathogenic microorganisms (Saati‐Santamaría et al., 2021a, 2021b). Although little information is available for *Ralstonia* in coleopterans, it is one of the few genera identified in the greater waxmoth (*Galleria mellonella*) believed to be a permanent fixture, present in egg/larval/adult stages (Gohl et al., 2022; Pilling et al., 2025). *Stenotrophomonas* are increasingly recognized as key gut symbionts in many insect species. They form key mutualistic relationships by breaking down environmental toxins, including pesticides (Duan et al., 2025; Guan et al., 2008). Moreover, network analyses revealed several non‐random associations among genera that were particularly evident for the larval stage. The co‐occurrence of these bacteria suggests these communities have some structural similarities across their geographical range above and beyond being transiently present. Additional research is needed to discriminate between transient and persistent bacteria harboured by *H. bicolor*.

In conclusion, our study provides one of the most comprehensive investigations into the microbiota harboured by coleopterans. Multiple factors contributed to the plasticity of the *H. bicolor* bacteriome, including developmental stage, host genetics and sampling location. This line of research has the potential to produce environmentally friendly and sustainable management outcomes, such as the development of plant‐incorporated semiochemicals and microbial insecticides (Qadri et al., 2020). Further, our study provides evidence for cryptic forms of *H. bicolor* that differ in their reproductive strategy and bacteriome composition. As *H. bicolor* is a significant agricultural pest during its larval stages, these findings could have implications to integrated pest management. Research is currently being undertaken to develop sex pheromones for the main pest wireworm species in Canada (Gries et al., 2021; Gries et al., 2022), and given the lack of males, such pheromones would be ineffective for surveillance and management approaches of the parthenogenetic form of *H. bicolor* (Catton et al., 2021). Similarly, pest control strategies that target a single sex are becoming more prevalent (e.g., male sterility), but are impractical for asexually reproduction populations. Also of note for management, control measures that disrupt the microbiota (e.g., sublethal insecticides or entomopathogens) could potentially allow pathogenic bacteria already present in the wireworm microbiome to overwhelm the wireworm immune system, thereby augmenting their effect (Ericsson et al., 2007; Kabaluk et al., 2017). From an evolutionary perspective, parthenogenetic populations may be endosymbiont‐mediated, but require future studies to characterize the roles (if any) the *H. bicolor* bacteriome plays in reproductive strategy.

## AUTHOR CONTRIBUTIONS

**Ivan Drahun:** Conceptualization; investigation; methodology; visualization; writing – review and editing. **Augustine Chukwunta:** Formal analysis; software. **Adeniyi Ayodele:** Formal analysis. **Ben G. Pilling:** Methodology; formal analysis; writing – review and editing; visualization. **Willem G. van Herk:** Conceptualization; writing – review and editing; data curation; investigation; project administration; resources. **Bryan J. Cassone:** Conceptualization; investigation; funding acquisition; writing – original draft; methodology; validation; software; formal analysis; project administration; data curation; supervision; resources.

## FUNDING INFORMATION

This research was funded by the Natural Sciences and Engineering Research Council of Canada (NSERC) Discovery grant awarded to Bryan Cassone (RGPIN‐2023‐04126).

## CONFLICT OF INTEREST STATEMENT

The authors declare there are no conflicts of interest.

## Supporting information


**Figure S1.** Visualization of the correlation between the number of significant bacterial genera identified and geographic distance between localities for both beetles (*R* ^2^ = 0.698; *p* = 0.025) and wireworms (*R* ^2^ = 0.576, *p* = 0.232).

**Table S1.** Sample information and sequencing statistics (i.e., number of reads remaining after filtration steps) for *Hypnoidus bicolor* beetles and wireworm samples subjected to 16S rRNA gene sequencing.

**Table S2.** Group differences in the structure and composition of bacteriomes of *Hypnoidus bicolor* across all sampling localities. Displayed are significant genera (or next hierarchical classification that could be resolved) across sampling localities based on ANCOM‐BC2 analysis (*q* < 0.05).

## Data Availability

Raw data are deposited at the NCBI SRA database under the accession number PRJNA1100600 (SUB14380285) (Cassone, 2026). Available from: https://www.ncbi.nlm.nih.gov/sra.

## References

[imb70054-bib-0001] Adams, A.S. , Aylward, F.O. , Adams, S.M. , Erbilgin, N. , Aukema, B.H. , Currie, C.R. et al. (2013) Mountain pine beetles colonizing historical and naive host trees are associated with a bacterial community highly enriched in genes contributing to terpene metabolism. Applied and Environmental Microbiology, 79, 3468–3475. Available from: 10.1128/AEM.00068-13 PMC3648045 23542624

[imb70054-bib-0002] Aguín‐Pombo, D. & Kuznetsova, V.G. (2023) True parthenogenesis and female‐biased sex ratios in Cicadomorpha and Fulgoromorpha (Hemiptera, Auchenorrhyncha). Insects, 14, 820. Available from: 10.3390/insects14100820 PMC10607665 37887832

[imb70054-bib-0003] Aikawa, T. , Maehara, N. , Ichihara, Y. , Masuya, H. , Nakamura, K. & Anbutsu, H. (2022) Cytoplasmic incompatibility in the semivoltine longicorn beetle *Acalolepta fraudatrix* (coleoptera: Cerambycidae) double infected with *Wolbachia* . PLoS One, 14, e0261928. Available from: 10.1371/journal.pone.0261928 PMC8759696 35030199

[imb70054-bib-0004] Andrews, K.R. , Gerritsen, A. , Rashed, A. , Crowder, D.W. , Rondon, S.I. , van Herk, W.G. et al. (2020) Wireworm (Coleoptera: Elateridae) genomic analysis reveals putative cryptic species, population structure, and adaptation to pest control. Communications Biology, 3, 489. Available from: 10.1038/s42003-020-01169-9 PMC7477237 32895437

[imb70054-bib-0005] Arai, H. , Legeai, F. , Kageyama, D. , Sugio, A. & Simon, J.‐C. (2024) Genomic insights into *Spiroplasma* endosymbionts that induce male‐killing and protective phenotypes in the pea aphid. FEMS Microbiology Letters, 371, fnae027. Available from: 10.1093/femsle/fnae027 38632047

[imb70054-bib-0006] Barsics, F. , Haubruge, E. & Verheggen, F.J. (2013) Wireworms' management: an overview of the existing methods, with particular regards to *Agriotes* spp. (Coleoptera: Elateridae). Insects, 4, 117–152. Available from: 10.3390/insects4010117 PMC4553433 26466799

[imb70054-bib-0007] Baselga, A. & Orme, C.D. (2012) Betapart: an R package for the study of beta diversity. Methods in Ecology and Evolution, 3, 808–812. Available from: 10.1111/j.2041-210X.2012.00224.x

[imb70054-bib-0008] Boelsen, L.K. , Dunne, E.M. , Mika, M. , Eggers, S. , Nguyen, C.D. , Ratu, F.T. et al. (2019) The association between pneumococcal vaccination, ethnicity, and the nasopharyngeal microbiota of children in Fiji. Microbiome, 7, 106. Available from: 10.1186/s40168-019-0716-4 PMC6636143 31311598

[imb70054-bib-0009] Bolyen, E. , Rideout, J.R. , Dillon, M.R. , Bokulich, N.A. , Abnet, C.C. , al‐Ghalith, G.A. et al. (2019) Reproducible, interactive, scalable and extensible microbiome data science using QIIME 2. Nature Biotechnology, 37, 852–857. 10.1038/s41587-019-0209-9 PMC7015180 31341288

[imb70054-bib-0010] Bozorov, T.A. , Toshmatov, Z.O. , Kahar, G. , Zhang, D. , Shao, H. & Gafforov, Y. (2021) Wild apple‐associated fungi and bacteria compete to colonize the larval gut of an invasive wood‐borer *Agrilus mali* in Tianshan forests. Frontiers in Microbiology, 12, 743831. Available from: 10.3389/fmicb.2021.743831 PMC8554297 34721341

[imb70054-bib-0011] Braglia, C. , Cutajar, S. , Magagnoli, S. , Asciano, D. , Burgio, G. , Di Gioia, D. et al. (2025) The ground beetle *Poecilus* (Carabidae) gut microbiome and its functionality. Microbial Ecology, 88, 83. Available from: 10.1007/s00248-025-02579-0 PMC12310908 40736560

[imb70054-bib-0012] Brandl, M.A. , Schumann, M. , Przyklenk, M. , Patel, A. & Vidal, S. (2017) Wireworm damage reduction in potatoes with an attract‐and‐kill strategy using *Metarhizium brunneum* . Journal of Pest Science, 90, 479–493. Available from: 10.1007/s10340-016-0824-x

[imb70054-bib-0013] Bray, J. & Curtis, J.T. (1957) An ordination of the upland forest communities of southern Wisconsin. Ecological Monographs, 27, 325–349.

[imb70054-bib-0014] Brooks, A.R. (1961) Adult Elateridae of southern Alberta, Saskatchewan and Manitoba (Coleoptera). The Canadian Entomologist, 92, 5–63. Available from: 10.4039/entm9320fv

[imb70054-bib-0015] Callahan, B. , McMurdie, P. , Rosen, M. et al. (2016) DADA2: high‐resolution sample inference from Illumina amplicon data. Nature Methods, 13, 581–583. Available from: 10.1038/nmeth.3869 PMC4927377 27214047

[imb70054-bib-0016] Cassone, B.J. (2026) Microbiomes of adult and larval H. Bicolor clade a and B across multiple populations encompassing all three prairie provinces. Bethesda, Maryland: NCBI Sequence Read Archive (SRA). Accession Numbers: SRR28708245 ‐ SRR28708313. https://www.ncbi.nlm.nih.gov/sra

[imb70054-bib-0017] Catton, H. , van Herk, W.G. , Saguez, J. & Svendsen, E. (2021) Guide to pest wireworms in Canadian prairie field crop production. Lethbridge, Alberta, Canada: Agriculture and Agri‐Food Canada.

[imb70054-bib-0018] Chiang, M.‐R. & Shelomi, M. (2023) Anatomical changes of the beetle digestive tract during metamorphosis correspond to dietary changes. Journal of Morphology, 284, e21575. Available from: 10.1002/jmor.21575 36826409

[imb70054-bib-0019] Chong, J. , Liu, P. , Zhou, G. & Xia, J. (2020) Using MicrobiomeAnalyst for comprehensive statistical, functional, and meta‐analysis of microbiome data. Nature Protocols, 15, 799–821. Available from: 10.1038/s41596-019-0264-1 31942082

[imb70054-bib-0020] Crippen, T.L. , Singh, B. , Anderson, R.C. & Sheffield, C.L. (2022) Management practices affecting lesser mealworm larvae (*Alphitobius diaperinus*) associated microbial community in a broiler house and after relocating with the litter into pastureland. Frontiers in Microbiology, 13, 875930. Available from: 10.3389/fmicb.2022.875930 PMC9283091 35847098

[imb70054-bib-0021] Danismazoglu, M. , Demir, İ. , Sevim, A. , Demirbag, Z. & Nalcacioglu, R. (2012) An investigation on the bacterial flora of *Agriotes lineatus* (coleoptera: Elateridae) and pathogenicity of the flora members. Crop Protection, 40, 1–7. Available from: 10.1016/j.cropro.2012.04.012

[imb70054-bib-0022] Davis, N.M. , Proctor, D.M. , Holmes, S.P. , Relman, D.A. & Callahan, B.J. (2018) Simple statistical identification and removal of contaminant sequences in marker‐gene and metagenomics data. Microbiome, 6, 226. Available from: 10.1186/s40168-018-0605-2 PMC6298009 30558668

[imb70054-bib-0023] Dhariwal, A. , Chong, J. , Habib, S. , King, I.L. , Agellon, L.B. & Xia, J. (2017) MicrobiomeAnalyst: a web‐based tool for comprehensive statistical, visual and meta‐analysis of microbiome data. Nucleic Acids Research, 45, W180–W188. Available from: 10.1093/nar/gkx295 PMC5570177 28449106

[imb70054-bib-0024] Drahun, I. , Hunt, K.A. , van Herk, W.G. & Cassone, B.J. (2022) Evaluation of standardized bait trapping approaches and climatic factors that influence wireworm catch in the Canadian prairies. Journal of Economic Entomology, 115, 773–782. Available from: 10.1093/jee/toac037 35385052

[imb70054-bib-0025] Drahun, I. , Morrison, K. , Poole, E.A. , van Herk, W.G. & Cassone, B.J. (2025) Characterisation of the bacteriomes harboured by major wireworm pest species in the Canadian prairies. Insect Molecular Biology, 34, 203–217. Available from: 10.1111/imb.12962 PMC11705518 39381854

[imb70054-bib-0026] Drahun, I. , Poole, E.A. , Hunt, K.A. , van Herk, W.G. , LeMoine, C.M. & Cassone, B.J. (2023) Seasonal turnover and insights into the overwintering biology of wireworms (Coleoptera: Elateridae) in the Canadian prairies. Pest Management Science, 79, 526–536. Available from: 10.1002/ps.7222 36196672

[imb70054-bib-0027] Drahun, I. , Rutherford, P.L. , van Herk, W.G. & Cassone, B.J. (2024) Wireworm feeding behaviours: the capacity of *Hypnoidus bicolor* to damage soybean under different environmental conditions. Arthropod‐Plant Interactions, 18, 129–139. Available from: 10.1007/s11829-023-10009-6

[imb70054-bib-0028] Drahun, I. , Wiebe, K.F. , Gohl, P. , Koloski, C.W. , Koiter, A.J. , van Herk, W.G. et al. (2021) Three years of surveillance associates agro‐environmental factors with wireworm infestations in Manitoba, Canada. Pest Management Science, 78, 369–378. Available from: 10.1002/ps.6650 34538023

[imb70054-bib-0029] Drahun, I. , Wiebe, K.F. , Koloski, C.W. , van Herk, W.G. & Cassone, B.J. (2021) Genetic structure and population demographics of *Hypnoidus bicolor* (Coleoptera: Elateridae) in the Canadian prairies. Pest Management Science, 77, 2282–2291. Available from: 10.1002/ps.6255 33421259

[imb70054-bib-0030] Duan, Y. , Luo, D. , Jian, B. , Wu, L. , Yang, H. , Hu, C. et al. (2025) The intestinal *Stenotrophomonas maltophilia* ZG‐GX enhances the resistance of *Spodoptera frugiperda* to beta‐cypermethrin and decreases the amino acid levels. Microbiology Spectrum, 14, e0315025. Available from: 10.1128/spectrum.03150-25 PMC12772350 41251600

[imb70054-bib-0031] Duran, D.P. , Herrmann, D.P. , Roman, S.J. , Gwiazdowski, R.A. , Drummond, J.A. , Hood, G.R. et al. (2019) Cryptic diversity in the north American Dromochorus tiger beetles (Coleoptera: Carabidae: Cicindelinae): a congruence‐based method for species discovery. Zoological Journal of the Linnean Society, 186, 250–285. Available from: 10.1093/zoolinnean/zly035

[imb70054-bib-0032] Ebert, K.M. , Arnold, W.G. , Ebert, P.R. & Merritt, D.J. (2021) Hindgut microbiota reflects different digestive strategies in dung beetles (Coleoptera: Scarabaeidae: Scarabaeinae). Applied and Environmental Microbiology, 87, e02100‐20. Available from: 10.1128/AEM.02100-20 PMC8090880 33355113

[imb70054-bib-0033] Ericsson, J.D. , Todd Kabaluk, J. , Goettel, M.S. & Myers, J.H. (2007) Spinosad interacts synergistically with the insect pathogen Metarhizium anisopliae against the exotic wireworms *Agriotes lineatus* and *Agriotes obscurus* (coleoptera: Elateridae). Journal of Economic Entomology, 100, 31–38. Available from: 10.1093/jee/100.1.31 17370806

[imb70054-bib-0034] Estes, A.M. , Hearn, D.J. , Snell‐Rood, E.C. , Feindler, M. , Feeser, K. , Abebe, T. et al. (2013) Brood ball‐mediated transmission of microbiome members in the dung beetle, *Onthophagus taurus* (Coleoptera: Scarabaeidae). PLoS One, 8, e79061. Available from: 10.1371/journal.pone.0079061 PMC3815100 24223880

[imb70054-bib-0035] Etzler, F.E. (2013) Identification of economic wireworms using traditional and molecular methods. Master of Science Thesis, Montana State University.

[imb70054-bib-0036] Finkel, O.M. , Burch, A.Y. , Elad, T. , Huse, S.M. , Lindow, S.E. , Post, A.F. et al. (2012) Distance‐decay relationships partially determine diversity patterns of phyllosphere bacteria on tamrix trees across the Sonoran desert. Applied and Environmental Microbiology, 78, 6187–6193. Available from: 10.1128/AEM.00888-12 PMC3416633 22752165

[imb70054-bib-0037] Folmer, O. , Black, M. , Hoeh, W. , Lutz, R. & Vrijenhoek, R. (1994) DNA primers for amplification of mitochondrial cytochrome *c* oxidase subunit I from diverse metazoan invertebrates. Molecular Marine Biology and Biotechnology, 3, 294–299. 7881515

[imb70054-bib-0038] Friedman, J. & Alm, E.J. (2012) Inferring correlation networks from genomic survey data. PLoS Computational Biology, 8, e1002687. Available from: 10.1371/journal.pcbi.1002687 PMC3447976 23028285

[imb70054-bib-0039] Gedling, C.R. , Smith, C.M. , LeMoine, C.M.R. & Cassone, B.J. (2018) The Mexican bean beetle (*Epilachna varivestis*) regurgitome and insights into beetle‐borne virus specificity. PLoS One, 13, e0192003. Available from: 10.1371/journal.pone.0192003 PMC5788362 29377955

[imb70054-bib-0040] Giglio, A. , Vommaro, M.L. , Gionechetti, F. & Pallavicini, A. (2021) Gut microbial community response to herbicide exposure in a ground beetle. Journal of Applied Entomology, 145, 986–1000. Available from: 10.1111/jen.12919

[imb70054-bib-0041] Glen, R. , King, K. & Arnason, A.P. (1943) The identification of wireworms of economic importance in Canada. Canadian Journal of Research, 21, 358–387. Available from: 10.1139/cjr43d-030

[imb70054-bib-0042] Gohl, P. , LeMoine, C.M.R. & Cassone, B.J. (2022) Diet and ontogeny drastically alter the larval microbiome of the invertebrate model *Galleria mellonella* . Canadian Journal of Microbiology, 68, 594–604. Available from: 10.1139/cjm-2022-0058 35863073

[imb70054-bib-0043] Gries, R. , Alamsetti, S.K. , van Herk, W.G. , Catton, H.A. , Meers, S. , Lemke, E. et al. (2021) Limoniic acid‐major component of the sex pheromones of the click beetles *Limonius canus* and *L. californicus* . Journal of Chemical Ecology, 47, 123–133. Available from: 10.1007/s10886-020-01241-y 33606109

[imb70054-bib-0044] Gries, R. , van Herk, W. , Alamsetti, S.K. , Catton, H. , Meers, S. , Otani, J. et al. (2022) (Z, E)‐α‐Farnesene–sex pheromone component of female click beetle *Selatosomus aeripennis destructor* with intra‐and inter‐sexual communication function. Entomologia Experimentalis et Applicata, 170, 344–351. Available from: 10.1111/eea.13142

[imb70054-bib-0045] Guan, S. , Ji, C. , Zhou, T. , Li, J. , Ma, Q. & Niu, T. (2008) Aflatoxin B1 degradation by *Stenotrophomonas maltophilia* and other microbes selected using coumarin medium. International Journal of Molecular Sciences, 9, 1489–1503. Available from: 10.3390/ijms9081489 PMC2635738 19325817

[imb70054-bib-0046] Hammer, T.J. & Bowers, M.D. (2015) Gut microbes may facilitate insect herbivory of chemically defended plants. Oecologia, 179, 1–14. Available from: 10.1007/s00442-015-3327-1 25936531

[imb70054-bib-0047] Hebert, P.D.N. , Cywinska, A. , Ball, S.L. & Dewaard, J.R. (2003) Biological identifications through DNA barcodes. Proceedings of the Biological Sciences, 270, 313–321. Available from: 10.1098/rspb.2002.2218 PMC1691236 12614582

[imb70054-bib-0048] Hendrichs, J. , Vera, T.M. , De Meyer, M. & Clarke, A.R. (2015) Resolving cryptic species complexes of major tephritid pests. ZooKeys, 540, 5–39. Available from: 10.3897/zookeys.540.9656 PMC4714062 26798252

[imb70054-bib-0049] Hughes, J.B. , Hellmann, J.J. , Ricketts, T.H. & Bohannan, B.J. (2001) Counting the uncountable: statistical approaches to estimating microbial diversity. Applied and Environmental Microbiology, 67, 4399–4406. Available from: 10.1128/AEM.67.10.4399-4406.2001 PMC93182 11571135

[imb70054-bib-0050] Hurst, G.D. & Jiggins, F.M. (2000) Male‐killing bacteria in insects: mechanisms, incidence, and implications. Emerging Infectious Diseases, 6, 329–336. Available from: 10.3201/eid0604.000402 PMC2640894 10905965

[imb70054-bib-0051] Hurst, G.D.D. , Schulenburg, H.G. , Majerus, T.M.O. , Zakharov, I.A. & Baungaard, J. (1999) Invasion of one insect species, *Adalia bipunctata*, by two different male‐killing bacteria. Insect Molecular Biology, 8, 133–139. Available from: 10.1046/j.1365-2583.1999.810133.x 9927182

[imb70054-bib-0052] Ibarra‐Juarez, L.A. , Desgarennes, D. , Vázquez‐Rosas‐Landa, M. , Villafan, E. , Alonso‐Sánchez, A. , Ferrera‐Rodríguez, O. et al. (2018) Impact of rearing conditions on the ambrosia beetle's microbiome. Life (Basel), 8, 63. Available from: 10.3390/life8040063 PMC6316638 30551580

[imb70054-bib-0054] Jiggins, F.M. , Hurst, G.D.D. , Jiggins, C.D. , . v. d Schulenburg, J.H.G. & Majerus, M.E.N. (2000) The butterfly *Danaus chrysippus* is infected by a male‐killing *Spiroplasma* bacterium. Parasitology, 120, 439–446. Available from: 10.1017/s0031182099005867 10840973

[imb70054-bib-0055] Kabaluk, J.T. , Li‐Leger, E. & Nam, S. (2017) *Metarhizium brunneum* ‐ an enzootic wireworm disease and evidence for its suppression by bacterial symbionts. Journal of Invertebrate Pathology, 150, 82–87. Available from: 10.1016/j.jip.2017.09.012 28919016

[imb70054-bib-0056] Kageyama, D. , Nishimura, G. , Hoshizaki, S. & Ishikawa, Y. (2002) Feminizing *Wolbachia* in an insect, *Ostrinia furnacalis* (Lepidoptera: Crambidae). Heredity, 88, 444–449. Available from: 10.1038/sj.hdy.6800077 12180086

[imb70054-bib-0057] Kiefer, J.S.T. , Schmidt, G. , Krüsemer, R. , Kaltenpoth, M. & Engl, T. (2022) *Wolbachia* causes cytoplasmic incompatibility but not male‐killing in a grain pest beetle. Molecular Ecology, 31, 6570–6587. Available from: 10.1111/mec.16717 36201377

[imb70054-bib-0058] Kleespies, R.G. , Ritter, C. , Zimmermann, G. , Burghause, F. , Feiertag, S. & Leclerque, A. (2012) A survey of microbial antagonists of *Agriotes* wireworms from Germany and Italy. Journal of Pest Science, 86, 99–106. Available from: 10.1007/s10340-012-0447-9

[imb70054-bib-0059] Kucuk, R.A. , Campbell, B.J. , Lyon, N.J. , Shelby, E.A. & Caterino, M.S. (2023) Gut bacteria of adult and larval *Cotinis nitida* Linnaeus (Coleoptera: Scarabaeidae) demonstrate community differences according to respective life stage and gut region. Frontiers in Microbiology, 14, 1185661. Available from: 10.3389/fmicb.2023.1185661 PMC10362445 37485511

[imb70054-bib-0060] Labrie, G. , Gagnon, A.E. , Vanasse, A. , Latraverse, A. & Tremblay, G. (2020) Impacts of neonicotinoid seed treatments on soil‐dwelling pest populations and agronomic parameters in corn and soybean in Quebec (Canada). PLoS One, 15, e0229136. Available from: 10.1371/journal.pone.0229136 PMC7043745 32101547

[imb70054-bib-0061] Lacey, L.A. , Unruh, T.R. , Simkins, H. & Thomsen‐Archer, K. (2007) Gut bacteria associated with the Pacific coast wireworm, *Limonius canus*, inferred from 16 s rDNA sequences and their implications for control. Phytoparasitica, 35, 479–489. Available from: 10.1007/BF03020607

[imb70054-bib-0062] Leclerque, A. , Kleespies, R.G. , Ritter, C. , Schuster, C. & Feiertag, S. (2011) Genetic and electron‐microscopic characterization of '*Rickettsiella agriotidis*', a new *Rickettsiella* pathotype associated with wireworm, *Agriotes* sp. (coleoptera: Elateridae). Current Microbiology, 63, 158–163. Available from: 10.1007/s00284-011-9958-5 21638041

[imb70054-bib-0063] Li, H. , Jiang, Z. , Zhou, J. , Liu, X. , Zhang, Y. & Chu, D. (2023) Ecological factors associated with the distribution of *Bemisia tabaci* cryptic species and their facultative endosymbionts. Insects, 14, 252. Available from: 10.3390/insects14030252 PMC10053707 36975937

[imb70054-bib-0064] Li, X. , Fang, T. , Gao, T. , Gui, H. , Chen, Y. , Zhou, L. et al. (2024) Widespread presence of gut bacterium *Glutamicibacter ectropisis* sp. nov. confers enhanced resistance to the pesticide bifenthrin in tea pests. Sci Total Environ, 955, 176784. Available from: 10.1016/j.scitotenv.2024.176784 39414054

[imb70054-bib-0065] Li, Z. , Huang, S. , He, X. , Ma, H. , Zhou, X. , Lin, H. et al. (2022) Specific enriched *Acinetobacter* in *camellia* weevil gut facilitate the degradation of tea saponin: inferred from bacterial genomic and transcriptomic analyses. Microbiol Spectr, 10, e0227222. Available from: 10.1128/spectrum.02272-22 PMC9769793 36413019

[imb70054-bib-0066] Lin, H. & Peddada, S.D. (2020) Analysis of compositions of microbiomes with bias correction. Nature Communications, 11, 3514. Available from: 10.1038/s41467-020-17041-7 PMC7360769 32665548

[imb70054-bib-0067] Lozupone, C.A. & Knight, R. (2008) Species divergence and the measurement of microbial diversity. FEMS Microbiology Reviews, 32, 557–578. Available from: 10.1111/j.1574-6976.2008.00111.x PMC2443784 18435746

[imb70054-bib-0068] Lu, Q.‐C. , Yu, J.‐M. , Liu, H.‐L. , Wu, X.‐L. , Wei, S.‐J. , Lei, M. et al. (2024) Stable composition of gut microbiome in the Asian ladybeetle *Coccinella septempunctata* reared on natural and artificial diets. Scientific Reports, 14, 71. Available from: 10.1038/s41598-023-49885-6 PMC10761721 38168578

[imb70054-bib-0069] Łukasik, P. , van Asch, M. , Guo, H. , Ferrari, J. & Godfray, H.C.J. (2013) Unrelated facultative endosymbionts protect aphids against a fungal pathogen. Ecology Letters, 16, 214–218. Available from: 10.1111/ele.12031 23137173

[imb70054-bib-0070] Magagnoli, S. , Alberoni, D. , Baffoni, L. , Martini, A. , Marini, F. , Di Gioia, D. et al. (2022) The ground beetle *Pseudoophonus rufipes* gut microbiome is influenced by the farm management system. Scientific Reports, 12, 22638. Available from: 10.1038/s41598-022-25408-7 PMC9805440 36587034

[imb70054-bib-0071] Martin, M. (2011) Cutadapt removes adapter sequences from high‐throughput sequencing reads. EMBnet.Journal, 17, 10–12. Available from: 10.14806/ej.17.1.200

[imb70054-bib-0072] Mayoral‐Peña, Z. , Lázaro‐Vidal, V. , Fornoni, J. , Álvarez‐Martínez, R. & Garrido, E. (2022) Studying plant–insect interactions through the analyses of the diversity, composition, and functional inference of their bacteriomes. Microorganisms, 11, 40. 10.3390/microorganisms11010040 PMC9863603 36677331

[imb70054-bib-0073] Mohammed, W.S. , Ziganshina, E.E. , Shagimardanova, E.I. , Gogoleva, N.E. & Ziganshin, A.M. (2018) Comparison of intestinal bacterial and fungal communities across various xylophagous beetle larvae (Coleoptera: Cerambycidae). Scientific Reports, 8, 10073. Available from: 10.1038/s41598-018-27342-z PMC6030058 29968731

[imb70054-bib-0074] Moldovan, O.T. , Carrell, A.A. , Bulzu, P.A. , Levei, E. , Bucur, R. , Sitar, C. et al. (2023) The gut microbiome mediates adaptation to scarce food in coleoptera. Environmental Microbiomes, 18, 80. Available from: 10.1186/s40793-023-00537-2 PMC10644639 37957741

[imb70054-bib-0075] Muratore, M. , Prather, C. & Sun, Y. (2020) The gut bacterial communities across six grasshopper species from a coastal tallgrass prairie. PLoS One, 15, e0228406. Available from: 10.1371/journal.pone.0228406 PMC6992175 31999781

[imb70054-bib-0076] Nor, I. , Engelstädter, J. , Duron, O. , Reuter, M. , Sagot, M.‐F. & Charlat, S. (2013) On the genetic architecture of cytoplasmic incompatibility: inference from phenotypic data. The American Naturalist, 182, E15–E24. Available from: 10.1086/670612 23778233

[imb70054-bib-0077] Paddock, K.J. , Finke, D.L. , Kim, K.S. , Sappington, T.W. & Hibbard, B.E. (2022) Patterns of microbiome composition vary across spatial scales in a specialist insect. Frontiers in Microbiology, 13, 898744. Available from: 10.3389/fmicb.2022.898744 PMC9201478 35722352

[imb70054-bib-0078] Palacio, M.F.H. , Montoya, O.I. , Saldamando, C.I. , García‐Bonilla, E. , Junca, H. , Cadavid‐Restrepo, G.E. et al. (2021) Dry and rainy seasons significantly alter the gut microbiome composition and reveal a key Enterococcussp. (Lactobacillales: Enterococcaceae) core component in *Spodoptera frugiperda* (lepidoptera: Noctuidae) corn strain from northwestern Colombia. Journal of Insect Science, 21, 10. Available from: 10.1093/jisesa/ieab076 PMC8567080 34734290

[imb70054-bib-0079] Parker, W.E. & Howard, J.J. (2002) The biology and management of wireworms (*Agriotes* spp.) on potato with particular reference to the U.K. Agricultural and Forest Entomology, 3, 85–98. Available from: 10.1046/j.1461-9563.2001.00094.x

[imb70054-bib-0080] Pilling, B.G. , Drahun, I. & Cassone, B.J. (2025) Discrimination between transient and persistent bacteria harbored in the digestive tract of larval *Galleria mellonella* . Entomologia Experimentalis et Applicata, 173, 978–991. Available from: 10.1111/eea.13598

[imb70054-bib-0081] Qadri, M. , Short, S. , Gast, K. , Hernandez, J. & Wong, A.C.‐N. (2020) Microbiome innovation in agriculture: development of microbial based tools for insect pest management. Front Sustain Food Syst, 4, 547751. Available from: 10.3389/fsufs.2020.547751

[imb70054-bib-0082] Quast, C. , Prusse, E. , Yilmaz, P. , Gerken, J. , Schweer, T. , Yarza, P. et al. (2013) The SILVA ribosomal RNA gene database project: improved data processing and web‐based tools. Nucleic Acids Research, 41, D590–D596. Available from: 10.1093/nar/gks1219 PMC3531112 23193283

[imb70054-bib-0083] R Core Team . (2025) R: a language and environment for statistical computing. Vienna, Austria: R foundation for statistical computing. https://www.R-project.org/

[imb70054-bib-0084] Rashed, A. & van Herk, W.G. (2024) Pest elaterids of North America: new insights and opportunities for management. Annual Review of Entomology, 69, 1–20. Available from: 10.1146/annurev-ento-120220-123249 37562049

[imb70054-bib-0085] Ritter, C. & Richter, E. (2013) Control methods and monitoring of *Agriotes* wireworms (Coleoptera: Elateridae). Journal of Plant Diseases and Protection, 120, 4–15. Available from: 10.1007/BF03356448

[imb70054-bib-0086] Rosenwald, L.C. , Sitvarin, M.I. & White, J.A. (2020) Endosymbiotic *Rickettsiella* causes cytoplasmic incompatibility in a spider host. Proceedings of the Biological Sciences, 287, 20201107. Available from: 10.1098/rspb.2020.1107 PMC7423472 32635864

[imb70054-bib-0087] Saati‐Santamaría, Z. , Rivas, R. , Kolařik, M. & García‐Fraile, P. (2021a) A new perspective of *pseudomonas*‐host interactions: distribution and potential ecological functions of the genus *pseudomonas* within the bark beetle holobiont. Biology, 10, 164. Available from: 10.3390/biology10020164 PMC7922261 33669823

[imb70054-bib-0088] Saati‐Santamaría, Z. , Rivas, R. , Kolařik, M. & García‐Fraile, P. (2021b) Associations between bark beetles and pseudomonas. Princeton, New Jersey, USA: Springer.

[imb70054-bib-0089] Schuster, C. , Kleespies, R.G. , Ritter, C. , Feiertag, S. & Leclerque, A. (2013) Multilocus sequence analysis (MLSA) of ‘*Rickettsiella agriotidis’*, an intracellular bacterial pathogen of *Agriotes* wireworms. Current Microbiology, 66, 1–9. Available from: 10.1007/s00284-012-0219-z 23007524

[imb70054-bib-0090] Scully, E.D. , Geib, S.M. , Carlson, J.E. , Tien, M. , McKenna, D. & Hoover, K. (2014) Functional genomics and microbiome profiling of the Asian longhorned beetle (*Anoplophora glabripennis*) reveal insights into the digestive physiology and nutritional ecology of wood feeding beetles. BMC Genomics, 15, 1096. Available from: 10.1186/1471-2164-15-1096 PMC4299006 25495900

[imb70054-bib-0091] Serrano, J.M. , Cook, R. , Headrick, H. & Cooper, W.R. (2023) Dietary history of click beetles and wireworms in the genus *Limonius* (Coleoptera: Elateridae) revealed by molecular gut content analysis. Environmental Entomology, 53, 173–179. Available from: 10.1093/ee/nvad114 38048447

[imb70054-bib-0092] Shao, Y. , Mason, C.J. & Felton, G.E. (2024) Toward an integrated understanding of the lepidoptera microbiome. Annual Review of Entomology, 69, 117–137. Available from: 10.1146/annurev-ento-020723-102548 37585608

[imb70054-bib-0093] Stibick, J.N.L. (1978) A revision of the Hypnoidinae of the world (Col. Elateridae). Part II. The Hypnoidinae of north and South America. The genera *Ascoliocerus*, *Desolakerrus*, *Margaiostus*, *Hypolithus* and *Hypnoidus* . Eos, 52, 309–386.

[imb70054-bib-0094] Stibick, J.N.L. (1979–1980) A revision of the Hypnoidinae of the world (Col. Elateridae). Part V. The Hypnoidinae of New Zealand, fossil Hypnoidinae, postscript and indices. Eos, 55‐56, 227–294.

[imb70054-bib-0095] Stork, N.E. (2018) How many species of insects and other terrestrial arthropods are there on earth? Annual Review of Entomology, 63, 31–45. Available from: 10.1146/annurev-ento-020117-043348 28938083

[imb70054-bib-0096] Suárez‐Moo, P. , Cruz‐Rosales, M. , Ibarra‐Laclette, E. , Desgarennes, D. , Huerta, C. & Lamelas, A. (2020) Diversity and composition of the gut microbiota in the developmental stages of the dung beetle *Copris incertus* say (Coleoptera, Scarabaeidae). Frontiers in Microbiology, 11, 1698. Available from: 10.3389/fmicb.2020.01698 PMC7393143 32793162

[imb70054-bib-0097] Takano, S. , Tuda, M. , Takasu, K. , Furuya, N. , Imamura, Y. , Kim, S. et al. (2017) Unique clade of alphaproteobacterial endosymbionts induces complete cytoplasmic incompatibility in the coconut beetle. Proceedings of the National Academy of Sciences of the United States of America, 114, 6110–6115. Available from: 10.1073/pnas.1618094114 PMC5468645 28533374

[imb70054-bib-0098] Tang, C. , Hu, X. , Tang, J. , Wang, L. , Liu, X. , Peng, Y. et al. (2024) The symbiont *Acinetobacter baumannii* enhances the insect host resistance to entomopathogenic fungus *Metarhizium anisopliae* . Communications Biology, 7, 1184. Available from: 10.1038/s42003-024-06779-1 PMC11412983 39300313

[imb70054-bib-0099] Tegtmeier, D. , Riese, C. , Geissinger, O. , Radek, R. & Brune, A. (2016) *Breznakia blatticola* gen. nov. sp. nov. and *Breznakia pachnodae* sp. nov., two fermenting bacteria isolated from insect guts, and emended description of the family Erysipelotrichaceae. Systematic and Applied Microbiology, 39, 319–329. Available from: 10.1016/j.syapm.2016.05.003 27270136

[imb70054-bib-0100] Traugott, M. , Benefer, C.M. , Blackshaw, R.P. , van Herk, W.G. & Vernon, R.S. (2015) Biology, ecology, and control of elaterid beetles in agricultural land. Annual Review of Entomology, 60, 313–334. Available from: 10.1146/annurev-ento-010814-021035 25341096

[imb70054-bib-0101] Tsuchida, T. , Koga, R. , Shibao, H. , Matsumoto, T. & Fukatsu, T. (2002) Diversity and geographic distribution of secondary endosymbiotic bacteria in natural populations of the pea aphid, *Acyrthosiphon pisum* . Molecular Ecology, 11, 2123–2135. Available from: 10.1046/j.1365-294x.2002.01606.x 12296954

[imb70054-bib-0102] Valdivia, C. , Newton, J.A. , Von Beeren, C. , O'Donnell, S. , Kronauer, D.J.C. , Russell, J.A. et al. (2023) Microbial symbionts are shared between ants and their associated beetles. Environmental Microbiology, 25, 3466–3483. Available from: 10.1111/1462-2920.16544 37968789

[imb70054-bib-0103] van der Kooi, C.J. , Matthey‐Doret, C. & Schwander, T. (2017) Evolution and comparative ecology of parthenogenesis in haplodiploid arthropods. Evolution Letters, 1, 304–316. Available from: 10.1002/evl3.30 PMC6121848 30283658

[imb70054-bib-0104] van Herk, W.G. & Vernon, R.S. (2014) Click beetles and wireworms (coleoptera: Elateridae) of Alberta, Saskatchewan, and Manitoba. In: Giberson, D.J. & Cárcamo, H.A. (Eds.) Arthropods of Canadian grasslands: biodiversity and systematics part 2, Vol. 4. Ottawa, Ontario: Biological Survey of Canada, pp. 87–117.

[imb70054-bib-0105] van Herk, W.G. , Vernon, R.S. , Goudis, L. & Mitchell, T. (2021) Broflanilide, a meta‐diamide insecticide seed treatment for protection of wheat and mortality of wireworms (*Agriotes obscurus*) in the field. Journal of Economic Entomology, 114, 161–173. Available from: 10.1093/jee/toaa239 33140830

[imb70054-bib-0106] van Herk, W.G. , Vernon, R.S. , Goudis, L. & Mitchell, T. (2022) Protection of potatoes and mortality of wireworms (*Agriotes obscurus*) with various application methods of Broflanilide, a novel meta‐diamide insecticide. Journal of Economic Entomology, 115, 1930–1946. Available from: 10.1093/jee/toac152 36222544

[imb70054-bib-0107] van Herk, W.G. , Vernon, R.S. , Labun, T.J. , Sevcik, M.H. & Schwinghamer, T.D. (2021) Distribution of pest wireworm (Coleoptera: Elateridae) species in Alberta, Saskatchewan, and Manitoba (Canada). Journal of Economic Entomology, 50, 663–672. Available from: 10.1093/ee/nvab006 33560338

[imb70054-bib-0108] van Herk, W.G. , Warren, R.L. & Bailey, T. (2023) Wireworm (Coleoptera: Elateridae) intoxication symptoms indicate the postharvest presence of broflanilide residues in soil collected from potato fields. Journal of Economic Entomology, 116, 1998–2008. Available from: 10.1093/jee/toad202 37942675

[imb70054-bib-0109] Vernon, R. & van Herk, W.G. (2017) Wireworm and flea beetle IPM in potatoes in Canada: implications for managing emergent problems in Europe. Potato Research, 60, 269–285.

[imb70054-bib-0110] Vernon, R.S. & van Herk, W.G. (2022) Chapter 7 ‐ wireworms as pests of potato. In: Alyokhin, A. , Rondon, S.I. & Gao, Y. (Eds.) Insect pests of potato (second edition). Cambridge, Massachusetts: Academic Press, pp. 103–148.

[imb70054-bib-0111] Vernon, R.S. , van Herk, W.G. , Clodius, M. & Tolman, J. (2016) Companion planting attract‐and‐kill method for wireworm management in potatoes. Journal of Pest Science, 89, 375–389. Available from: 10.1007/s10340-015-0707-6

[imb70054-bib-0112] Viswan, A. & Augustine, N. (2026) Insect gut microbiota as a reservoir of industrially relevant enzymes: a comprehensive review. Microbial Ecology, 89, 107. Available from: 10.1007/s00248-026-02726-1 PMC13171643 41936687

[imb70054-bib-0113] Wade, M.J. & Stevens, L. (1985) Microorganism mediated reproductive isolation in flour beetles (genus *Tribolium*). Science, 227, 527–528. Available from: 10.1126/science.3966160 3966160

[imb70054-bib-0114] Walter, G.H. (2003) Insect Pest management and ecological research. Cambridge, England: Cambridge University Press.

[imb70054-bib-0115] Wan, X. , Jiang, Y. , Cao, Y. , Sun, B. & Xiang, X. (2020) Divergence in gut bacterial community structure between male and female stag beetles Odontolabis fallaciosa (Coleoptera, Lucanidae). Animals, 10(12), 2352. Available from: 10.3390/ani10122352 PMC7764088 33317133

[imb70054-bib-0116] Wickham, H. (2016) ggplot2: elegant graphics for data analysis, 2nd edition. New York City NY: Springer‐Verlag New York. https://ggplot2.tidyverse.org

[imb70054-bib-0117] Wickham, H. , Averick, M. , Bryan, J. , Chang, W. , McGowan, L. , François, R. et al. (2019) Welcome to the tidyverse. Journal of Open Source Software, 4(43), 1686. Available from: 10.21105/joss.01686

[imb70054-bib-0118] Wickham, H. & Bryan, J. (2026) readxl: Read Excel Files. R package version 1.5.0. https://readxl.tidyverse.org

[imb70054-bib-0119] Wielkopolan, B. , Krawczyk, K. , Szabelska‐Beresewicz, A. & Obrepalska‐Steplowska, A. (2021) The structure of the cereal leaf beetle (*Oulema melanopus*) microbiome depends on the insect's developmental stage, host plant, and origin. Scientific Reports, 11, 20496. Available from: 10.1038/s41598-021-99411-9 PMC8516949 34650106

[imb70054-bib-0120] Yoshida, M. & Yoshii, M. (1959) Researches on the wireworm *Melanotus caudex* Lewis: XIV. On the bacteria living in the body of the wireworm. Japanese Journal of Applied Entomology and Zoology, 3, 190–194.

[imb70054-bib-0121] Yuan, X. , Zhang, X. , Liu, X. , Dong, Y. , Yan, Z. , Lv, D. et al. (2021) Comparison of gut bacterial communities of *Grapholita molesta* (Lepidoptera: Tortricidae) reared on different host plants. International Journal of Molecular Sciences, 22, 6843. Available from: 10.3390/ijms22136843 PMC8268091 34202141

[imb70054-bib-0122] Zacharuk, R.Y. (1958a) Note on two forms of *Hypolithus bicolor* Esch. (Coleoptera: Elateridae). The Canadian Entomologist, 90, 567–568. Available from: 10.4039/Ent90567-9

[imb70054-bib-0123] Zacharuk, R.Y. (1958b) Structures and functions of the reproductive systems of the prairie grain wireworm, *Ctenicera aeripennis destructor* (Brown) (Coleoptera: Elateridae). Canadian Journal of Zoology, 36, 725–751. Available from: 10.1139/z58-062

[imb70054-bib-0124] Zacharuk, R.Y. (1973a) Penetration of the cuticular layers of elaterid larvae by the fungus *Metarrhizium anisopliae*, and notes on a bacterial invasion. Journal of Invertebrate Pathology, 21, 101–106. Available from: 10.1016/0022-2011(73)90118-3

[imb70054-bib-0125] Zacharuk, R.Y. (1973b) Ultrastructural changes in the midgut epithelium of an elaterid larva (coleoptera) infected enterically with *Pseudomonas aeruginosa* . Canadian Journal of Microbiology, 19, 811–821. Available from: 10.1139/m73-131 4199437

[imb70054-bib-0126] Zacharuk, R.Y. (1974) Ultrastructural pathology of the epidermis of molting elaterid larvae (coleoptera) with a fungus and a bacterium in the ecdysial space. Journal of Invertebrate Pathology, 23, 13–21. Available from: 10.1016/0022-2011(74)90067-6

