# Supplementary material for: Bacteriomes, cryptic forms and evolution of a common wireworm pest species, Hypnoidus bicolor
Source: Insect Mol Biol. 2026 Jun 26;35(6):669–85. doi: 10.1111/imb.70054 (PMC13749587; doi:10.1111/imb.70054)
Supplement: Supplementary file 1 [file IMB-35-669-s001.docx]

**Fig. S1.** Visualization of the correlation between the number of significant bacterial genera identified and geographic distance between localities for both beetles (R^2^ = 0.698; p = 0.025) and wireworms (R^2^ = 0.576, p = .232).
