# Supplementary material for: Bacteriomes, cryptic forms and evolution of a common wireworm pest species, Hypnoidus bicolor
Source: Insect Mol Biol. 2026 Jun 26;35(6):669–85. doi: 10.1111/imb.70054 (PMC13749587; doi:10.1111/imb.70054)
Supplement: Supplementary file 2 [file IMB-35-669-s002.docx]

**Table S2**. Group differences in the structure and composition of bacteriomes of *Hypnoidus bicolor* across all sampling localities. Displayed are significant genera (or next hierarchical classification that could be resolved) across sampling localities based on ANCOM-BC2 analysis (q < 0.05).

Beetles

| **Taxon** | **W** | **q value** |
| --- | --- | --- |
| Alphaproteobacteria.1 | 328.129 | 2.32543E-21 |
| Rickettsiella | 212.103 | 5.42487E-19 |
| Jeotgalicoccus | 186.83 | 2.12497E-18 |
| Enterobacterales | 169.18 | 6.32811E-18 |
| Glutamicibacter | 134.101 | 1.24736E-16 |
| Acinetobacter | 105.651 | 2.6697E-15 |
| Sphingomonadaceae | 74.346 | 2.47551E-13 |
| Weissella | 63.8855 | 1.56238E-12 |
| Brevibacterium | 56.5468 | 6.65284E-12 |
| Methylobacterium-Methylorubrum | 47.9798 | 4.75589E-11 |
| Hephaestia | 44.4611 | 1.11232E-10 |
| Planococcaceae | 38.8977 | 4.47004E-10 |
| Brevundimonas | 39.004 | 4.47004E-10 |
| Burkholderia-Caballeronia-Paraburkholderia | 39.283 | 4.47004E-10 |
| Nitrosomonas | 37.3561 | 6.78741E-10 |
| Staphylococcus | 35.9264 | 1.01448E-09 |
| Stenotrophomonas | 35.4919 | 1.1035E-09 |
| Xanthobacteraceae | 33.758 | 1.88479E-09 |
| Ralstonia | 32.5856 | 2.70335E-09 |
| Lactobacillale | 29.141 | 9.34606E-09 |
| Psychrobacter | 28.0711 | 1.36247E-08 |
| Hyphomicrobium | 24.4017 | 6.20481E-08 |
| Escherichia-Shigella | 24.2065 | 6.48112E-08 |
| Nocardia | 23.1915 | 9.90161E-08 |
| Erysipelotrichaceae | 20.7248 | 3.15497E-07 |
| Comamonadaceae | 20.243 | 3.88093E-07 |
| Morganella | 17.9724 | 1.26581E-06 |
| Staphylococcaceae | 16.6282 | 2.64451E-06 |
| Nitrospira | 15.1557 | 6.25846E-06 |
| Arcticibacter | 15.0853 | 6.32347E-06 |
| Rhodococcus | 14.9998 | 6.45887E-06 |
| Micrococcaceae | 12.8329 | 2.63359E-05 |
| Streptomyces | 12.4857 | 3.25912E-05 |
| Orbaceae | 12.0692 | 4.26102E-05 |
| Enterococcus | 11.4146 | 6.50837E-05 |
| Dysgonomonas | 11.4169 | 6.50837E-05 |
| Solibacillus | 10.7948 | 0.000101304 |
| Lawsonella | 10.4137 | 0.00013267 |
| Cutibacterium | 10.3804 | 0.000132705 |
| Latilactobacillus | 10.1715 | 0.000152639 |
| Microbacteriaceae | 9.80635 | 0.000199697 |
| Dongia | 9.72214 | 0.000208761 |
| Tsukamurella | 9.62475 | 0.0002208 |
| Clostridium | 9.23725 | 0.000297434 |
| Rathayibacter | 9.14006 | 0.000315544 |
| Massilia | 8.1368 | 0.000735359 |
| Pseudomonas | 8.1135 | 0.000735359 |
| Spiroplasma | 7.80705 | 0.000948846 |
| Chryseobacterium | 7.37283 | 0.001386156 |
| Carnobacterium | 6.74427 | 0.002468896 |
| Roseomonas | 6.54926 | 0.002927272 |
| Rhizobiaceae | 6.36744 | 0.003434889 |
| 67-14 | 6.18004 | 0.00406304 |
| Segetibacter | 5.97732 | 0.004894033 |
| Janthinobacterium | 5.57163 | 0.007297219 |
| Pedobacter | 5.45211 | 0.008122553 |
| Mesorhizobium | 5.38791 | 0.008538493 |
| Adhaeribacter | 5.32307 | 0.00883472 |
| Rubrobacter | 5.32582 | 0.00883472 |
| Terrimonas | 5.15456 | 0.010396739 |
| Flavobacterium | 5.00832 | 0.011805374 |
| Bdellovibrio | 5.00611 | 0.011805374 |
| Lactococcus | 4.97475 | 0.012019424 |
| Enterobacteriaceae | 4.81066 | 0.014148658 |
| Bacillales | 4.43001 | 0.021215448 |
| Chitinophagaceae | 4.41787 | 0.021215448 |
| Nitrososphaeraceae | 4.26028 | 0.024969941 |
| Enhydrobacter | 4.1539 | 0.027771955 |
| Rickettsiaceae | 4.0402 | 0.031181866 |
| Alcaligenaceae | 3.93951 | 0.034525905 |
| Mycobacterium | 3.92316 | 0.03469086 |
| Sanguibacter-Flavimobilis | 3.8865 | 0.03569619 |
| Williamsia | 3.74379 | 0.04158903 |
| Microbacterium | 3.63589 | 0.04657925 |

**Wireworms**

| **Taxon** | **W** | **q value** |
| --- | --- | --- |
| Hephaestia | 62.31595143 | 1.4E-10 |
| Acinetobacter | 47.1029195 | 2E-09 |
| Vicinamibacteraceae | 46.68046945 | 2E-09 |
| Thermomonas | 41.22876253 | 7E-09 |
| Nitrosomonas | 37.29503667 | 1.9E-08 |
| Xanthomonadaceae | 32.36154174 | 8.5E-08 |
| Hyphomicrobium | 30.56874111 | 1.4E-07 |
| Blastocatellaceae | 24.64974953 | 1.4E-06 |
| Xanthobacteraceae | 24.02975782 | 1.6E-06 |
| Neorhizobium | 23.24108671 | 2E-06 |
| Microvirga | 22.67979629 | 2.4E-06 |
| Pseudoxanthomonas | 21.95873445 | 3.1E-06 |
| Chthoniobacter | 21.50597025 | 3.5E-06 |
| Arenimonas | 20.43275047 | 5.5E-06 |
| Herpetosiphon | 20.31606261 | 5.5E-06 |
| Sphingopyxis | 20.16079769 | 5.5E-06 |
| Sphingomonadaceae | 19.31491455 | 8E-06 |
| Rheinheimera | 18.54973704 | 1.1E-05 |
| Rhodocyclaceae | 17.87512923 | 1.5E-05 |
| Verrucomicrobium | 17.49913802 | 1.8E-05 |
| Yersiniaceae | 15.74316753 | 4.6E-05 |
| Taibaiella | 15.15438715 | 6.2E-05 |
| Sphingobium | 14.02937777 | 0.00012 |
| Luteolibacter | 13.48139274 | 0.00016 |
| Gordonia | 13.30594513 | 0.00017 |
| Actinomarinales | 12.93079182 | 0.00021 |
| Lysobacter | 12.69116341 | 0.00024 |
| Tsukamurella | 12.63620912 | 0.00024 |
| Verrucomicrobiaceae | 12.31562611 | 0.00028 |
| Oxalobacteraceae | 12.18090432 | 0.00028 |
| Pir4 | 12.1664855 | 0.00028 |
| Dermacoccaceae | 12.17790031 | 0.00028 |
| Sphingobacteriaceae | 11.69771271 | 0.00036 |
| Stenotrophomonas | 11.7211267 | 0.00036 |
| Peredibacter | 10.72832124 | 0.00066 |
| Chitinophagaceae | 10.7497094 | 0.00066 |
| Segniliparus | 10.73621122 | 0.00066 |
| Bacteriovorax | 10.24736912 | 0.00091 |
| Enterobacterales.1 | 10.04347398 | 0.00103 |
| Nocardioidaceae | 9.829111693 | 0.00118 |
| Cupriavidus | 9.746744122 | 0.00122 |
| Janthinobacterium | 9.434918354 | 0.00145 |
| Bacteroidia | 9.478988364 | 0.00145 |
| MB-A2-108 | 9.421056153 | 0.00145 |
| Pirellula | 9.371601924 | 0.00148 |
| Rugamonas | 9.269526286 | 0.00156 |
| Cellvibrio | 9.081493641 | 0.00177 |
| Sanguibacter-Flavimobilis | 9.017524397 | 0.00182 |
| Alkanindiges | 8.825528944 | 0.00207 |
| Patulibacter | 8.673130124 | 0.00228 |
| Chryseobacterium | 8.251441623 | 0.00309 |
| Deinococcus | 8.235328659 | 0.00309 |
| Fonticella | 8.221399252 | 0.00309 |
| Mesorhizobium | 8.125208889 | 0.00327 |
| Methylophilaceae | 8.099197622 | 0.00328 |
| Methylophilus | 7.987162484 | 0.00353 |
| Corynebacteriales | 7.812201662 | 0.004 |
| Phenylobacterium | 7.777533913 | 0.00405 |
| Clostridium.2 | 7.587586354 | 0.00466 |
| Williamsia | 7.525078151 | 0.00482 |
| Pseudoduganella | 7.473461778 | 0.00495 |
| SH-PL14 | 7.348438856 | 0.00541 |
| Jatrophihabitans | 7.307222798 | 0.00552 |
| Enterobacterales | 7.222044769 | 0.00584 |
| Streptomyces | 7.125662769 | 0.00624 |
| Pseudomonas | 7.033774632 | 0.00664 |
| Escherichia-Shigella | 6.870075751 | 0.00753 |
| Vicinamibacterales | 6.851205625 | 0.00754 |
| Adhaeribacter | 6.805826406 | 0.00773 |
| Caulobacter | 6.780424411 | 0.00779 |
| Algoriphagus | 6.751361172 | 0.00788 |
| Hyphomicrobiaceae | 6.632096418 | 0.00862 |
| Devosiaceae | 6.596914926 | 0.00877 |
| Ralstonia | 6.401440759 | 0.01027 |
| Sutterellaceae | 6.3838417 | 0.01029 |
| Variovorax | 6.261104727 | 0.01118 |
| Dongia | 6.26302746 | 0.01118 |
| Gitt-GS-136 | 6.218728989 | 0.01146 |
| Udaeobacter | 6.088499915 | 0.01272 |
| NS11-12 | 6.030053879 | 0.01324 |
| Nitrospira | 5.973525237 | 0.01376 |
| Bdellovibrio | 5.669284186 | 0.01794 |
| Pirellulaceae | 5.610749269 | 0.01871 |
| Rhodopseudomonas | 5.587653033 | 0.01888 |
| Terrimonas | 5.462047535 | 0.02097 |
| Longimicrobium | 5.335090612 | 0.02333 |
| Planctomycetale | 5.255028804 | 0.02431 |
| A4b | 5.275602122 | 0.02431 |
| Planctomycetes | 5.256786132 | 0.02431 |
| Duganella | 5.219331918 | 0.02486 |
| Pyrinomonadaceae | 5.161927185 | 0.02595 |
| Gammaproteobacteria | 5.137981507 | 0.02626 |
| Afipia | 5.101491756 | 0.02689 |
| Weeksellaceae | 5.053406368 | 0.02785 |
| Pseudonocardia | 4.920833207 | 0.03128 |
| Methylobacterium-Methylorubrum | 4.816242084 | 0.03422 |
| Lawsonella | 4.801088148 | 0.03437 |
| Burkholderia-Caballeronia-Paraburkholderia | 4.769637124 | 0.03507 |
| Rhodanobacteraceae.1 | 4.704988584 | 0.03629 |
| Pseudorhodoferax | 4.722784008 | 0.03629 |
| Alphaproteobacteria.1 | 0.032615572 | 0.03629 |
| Alsobacter | 4.685113064 | 0.03657 |
| Sphingobacterium | 4.628803547 | 0.03759 |
| Pirellulaceae.1 | 4.635422473 | 0.03759 |
| Saccharibacillus | 4.626883003 | 0.03759 |
| CCD24 | 4.480533926 | 0.04296 |
| Aquabacterium | 0.039100246 | 0.04467 |
| Nitrosospira | 4.402008424 | 0.04555 |
| Comamonadaceae | 4.358166853 | 0.04713 |
| Vicinamibacteraceae.2 | 4.314999295 | 0.04874 |
| Bosea | 4.301051497 | 0.04897 |
