# Supplementary material for: Bacteriomes, cryptic forms and evolution of a common wireworm pest species, Hypnoidus bicolor
Source: Insect Mol Biol. 2026 Jun 26;35(6):669–85. doi: 10.1111/imb.70054 (PMC13749587; doi:10.1111/imb.70054)
Supplement: Supplementary file 3 [file IMB-35-669-s003.docx]

**Table S1.** Sample information and sequencing statistics (i.e., number of reads remaining after filtration steps) for *Hypnoidus bicolor* beetles and wireworm samples subjected to 16S rRNA gene sequencing.

Beetle samples

| **Sample** | **Clade** | **Location** | **Total Reads** | **Quality Trim** | **Denoised and merged** | **Non-chimeric** |
| --- | --- | --- | --- | --- | --- | --- |
| PY1 | A | Altona | 67553 | 61003 | 57655 | 41396 |
| PY2 | A | Altona | 68425 | 58777 | 57068 | 54939 |
| PY3 | A | Altona | 91989 | 78385 | 74080 | 64047 |
| PY4 | A | Altona | 81859 | 67024 | 60143 | 51793 |
| PY7 | A | Altona | 113070 | 97104 | 86627 | 61628 |
| PY8 | A | Altona | 69958 | 57305 | 52460 | 39424 |
| PY9 | A | Altona | 75355 | 64143 | 56505 | 46365 |
| PY11 | A | Altona | 70776 | 58342 | 50327 | 47081 |
| PY13 | A | Altona | 82253 | 71172 | 68640 | 58425 |
| PZ3 | A | MedHat | 72788 | 61423 | 60035 | 56955 |
| PZ4 | A | MedHat | 70269 | 58611 | 55309 | 54613 |
| PZ5 | A | MedHat | 51210 | 42959 | 39522 | 29668 |
| PZ6 | A | MedHat | 45789 | 39501 | 38184 | 37542 |
| PZ7 | A | MedHat | 54277 | 48619 | 47046 | 46467 |
| PZ9 | A | MedHat | 48611 | 40244 | 38991 | 37782 |
| PZ10 | A | MedHat | 76398 | 66729 | 65148 | 61106 |
| U1 | B | Meota | 89256 | 79785 | 77407 | 76907 |
| U2 | B | Meota | 65030 | 57314 | 56261 | 55776 |
| U3 | B | Meota | 87768 | 73050 | 71322 | 68059 |
| U4 | B | Meota | 85865 | 78623 | 76206 | 75725 |
| U5 | B | Meota | 97417 | 85660 | 82050 | 80619 |
| V1 | B | Claresholm | 55465 | 47571 | 46807 | 45338 |
| V2 | B | Claresholm | 53088 | 42576 | 41221 | 39457 |
| V4 | B | Claresholm | 50809 | 40430 | 38285 | 37729 |
| V5 | B | Claresholm | 63925 | 56515 | 55602 | 54562 |
| V7 | B | Claresholm | 142522 | 125158 | 120892 | 118386 |
| V8 | B | Claresholm | 100072 | 84238 | 81532 | 79630 |
| V9 | B | Claresholm | 109289 | 98815 | 96065 | 95335 |
| X2 | A | Regina | 97682 | 75089 | 71362 | 64892 |
| X3 | A | Regina | 73322 | 61911 | 59922 | 53227 |
| X4 | A | Regina | 53755 | 45695 | 43372 | 36389 |
| X7 | A | Regina | 77792 | 57492 | 54499 | 53532 |
| X8 | A | Regina | 74723 | 64347 | 61116 | 53120 |
| X9 | A | Regina | 68010 | 56259 | 54931 | 53869 |

Wireworm samples

| **Sample** | **Clade** | **Location** | **Total Reads** | **Quality Trim** | **Denoised and merged** | **Non-chimeric** |
| --- | --- | --- | --- | --- | --- | --- |
| ES1 | A | Rivers | 60686 | 49084 | 47966 | 47670 |
| ES3 | A | Rivers | 48749 | 41354 | 40609 | 40468 |
| ES4 | A | Rivers | 11852 | 9327 | 8928 | 8928 |
| ES5 | A | Rivers | 52902 | 42166 | 41073 | 41057 |
| ES6 | B | Rivers | 49633 | 38893 | 37859 | 37618 |
| ES7 | B | Rivers | 57175 | 44772 | 43177 | 39138 |
| ES8 | B | Rivers | 34012 | 26555 | 25739 | 25739 |
| ES9 | B | Rivers | 39316 | 34066 | 33791 | 33791 |
| ES10 | B | Rivers | 45222 | 39221 | 38659 | 38659 |
| HS1 | A | Hamiota | 38798 | 31370 | 30754 | 30754 |
| HS2 | A | Hamiota | 37113 | 30777 | 30106 | 30057 |
| HS3 | A | Hamiota | 42579 | 35390 | 34597 | 34597 |
| HS4 | A | Hamiota | 45201 | 38124 | 37512 | 37426 |
| HS5 | A | Hamiota | 41041 | 34807 | 34376 | 34248 |
| HS6 | B | Hamiota | 44808 | 35767 | 34290 | 34277 |
| HS7 | B | Hamiota | 40688 | 34473 | 33878 | 33861 |
| HS8 | B | Hamiota | 40119 | 32803 | 32175 | 32175 |
| HS9 | B | Hamiota | 57952 | 46704 | 45590 | 45293 |
| HS10 | B | Hamiota | 35069 | 27931 | 27251 | 27251 |
| MS1 | A | Treesbank | 37126 | 30565 | 30298 | 30298 |
| MS2 | A | Treesbank | 32761 | 26582 | 26229 | 26229 |
| MS4 | A | Treesbank | 32105 | 26662 | 25958 | 25902 |
| MS5 | A | Treesbank | 44687 | 36551 | 35981 | 35965 |
| MS6 | B | Treesbank | 50676 | 41135 | 40013 | 38935 |
| MS7 | B | Treesbank | 45939 | 37892 | 37062 | 37062 |
| MS8 | B | Treesbank | 42617 | 34474 | 33834 | 33670 |
| MY1 | A | Neelin | 38598 | 29988 | 29010 | 28959 |
| MY2 | A | Neelin | 43364 | 33500 | 32439 | 32341 |
| MY3 | A | Neelin | 15361 | 12004 | 11624 | 11622 |
| MY4 | A | Neelin | 7221 | 5666 | 5361 | 5361 |
| MY5 | A | Neelin | 15175 | 12111 | 11724 | 11640 |
| MY6 | B | Neelin | 28981 | 24060 | 22992 | 22992 |
| MY8 | B | Neelin | 8507 | 6790 | 6680 | 6680 |
| MY9 | B | Neelin | 54405 | 44168 | 42901 | 42634 |
| MY10 | B | Neelin | 52600 | 42063 | 40852 | 40615 |
